# A zebrafish model of diabetic nephropathy shows hyperglycemia, proteinuria and activation of the PI3K/Akt pathway

**DOI:** 10.1242/dmm.050438

**Published:** 2024-05-29

**Authors:** Liqing Zang, Sei Saitoh, Kan Katayama, Weibin Zhou, Norihiro Nishimura, Yasuhito Shimada

**Affiliations:** ^1^ Graduate School of Regional Innovation Studies, Mie University, Tsu, Mie 514-8507, Japan; ^2^ Mie University Zebrafish Research Center, Tsu, Mie 514-8507, Japan; ^3^ Department of Biomedical Molecular Sciences (Anatomy II), Fujita Health University School of Medicine, Toyoake 470-1192, Japan; ^4^ Department of Cardiology and Nephrology, Mie University Graduate School of Medicine, Tsu, Mie 514-8507, Japan; ^5^ Division of Nephrology, Department of Medicine, Icahn School of Medicine at Mount Sinai, New York City, NY 10029-5674, USA; ^6^ Department of Integrative Pharmacology, Mie University Graduate School of Medicine, Tsu, Mie 514-8507, Japan

**Keywords:** Obesity, Type 2 diabetes, Diabetic nephropathy, Zebrafish, Proteinuria, Glomerular hypertrophy

## Abstract

Diabetic nephropathy (DN), as a complication of diabetes, is a substantial healthcare challenge owing to the high risk of morbidity and mortality involved. Although significant progress has been made in understanding the pathogenesis of DN, more efficient models are required to develop new therapeutics. Here, we created a DN model in zebrafish by crossing diabetic *Tg(acta1:dnIGF1R-EGFP)* and proteinuria-tracing *Tg(l-fabp::VDBP-GFP)* lines, named zMIR/VDBP. Overfed adult zMIR/VDBP fish developed severe hyperglycemia and proteinuria, which were not observed in wild-type zebrafish. Renal histopathology revealed human DN-like characteristics, such as glomerular basement membrane thickening, foot process effacement and glomerular sclerosis. Glomerular dysfunction was restored upon calorie restriction. RNA sequencing analysis demonstrated that DN zebrafish kidneys exhibited transcriptional patterns similar to those seen in human DN pathogenesis. Notably, the phosphatidylinositol 3-kinase (PI3K)/protein kinase B (Akt) signaling pathway was activated, a phenomenon observed in the early phase of human DN. In addition, metformin improved hyperglycemia and proteinuria in DN zebrafish by modulating Akt phosphorylation. Our results indicate that zMIR/VDBP fish are suitable for elucidating the mechanisms underlying human DN and could be a powerful tool for therapeutic discovery.

## Teaser

**Summary:** A zebrafish model of diabetic nephropathy (DN) shows key features of human DN, activation of the PI3K/Akt pathway and the protective role of metformin, which highlight the potential of this model for therapeutic discovery.

## INTRODUCTION

According to the International Diabetes Federation, the increasing prevalence of diabetes worldwide is a significant health concern (Sun et al., 2022). Approximately 537 million adults over 20 years of age have type 1 and type 2 diabetes mellitus (T1DM and T2DM, respectively), and this number is predicted to increase to 643 million by 2030 and 783 million by 2045. In 2021, diabetes caused 6.7 million deaths, with one death occurring every 5 s. The primary health effects of diabetes include microvascular complications such as diabetic retinopathy, nephropathy and neuropathy, which contribute to significant morbidity in patients with diabetes (Valencia and Florez, 2017). Diabetic nephropathy (DN), also known as diabetic kidney disease, is one of the most frequent and severe causes of death in patients with diabetes. DN has been identified as the leading cause of end-stage renal disease in high-income countries (Jha et al., 2013; Ritz et al., 2011) and is a leading cause of increasing renal replacement therapy worldwide (Locatelli et al., 2004). The prevalence of diabetes has increased globally. Moreover, patients with DN have a substantially higher risk of cardiovascular disease-related morbidity and mortality (Cosentino et al., 2020; Tziomalos and Athyros, 2015). Normal kidneys act as filters, allowing water or small molecules to pass through while retaining larger molecules, such as proteins. However, the filtering system in patients with DN is damaged, resulting in a leakage of proteins such as albumin into the urine. This excess protein in the urine is defined as proteinuria or albuminuria, a hallmark of disease severity, and is used clinically to guide therapies. DN is classified into microalbuminuria, characterized by urinary albumin excretion >20 μg/min, and macroalbuminuria, with urinary albumin excretion ≥200 μg/min. A typical renal pathological abnormality in DN is hypertrophy of kidney cells, including all glomerular and tubular cell types. Prolonged exposure to hyperglycemia leads to glomerular damage, characterized by the thickening of the glomerular basement membrane (GBM) (a crucial component of the filtration barrier). Additionally, this damage includes the expansion of the glomerular mesangium, the supportive tissue surrounding the glomerulus, and alterations in podocytes, the cells that enwrap the capillaries of the glomerulus. These changes disrupt normal glomerular filtration, leading to proteinuria and impaired kidney function (Jefferson et al., 2008). Despite the influence and seriousness of this disease, the pathogenic molecular mechanisms of DN are complex (Lin et al., 2018; Sugahara et al., 2021; Vallon and Komers, 2011; Van et al., 2017), and further studies are required for a better understanding.

Animal disease models are important to improve our understanding of human diseases, identify causative genes, and develop effective and novel therapies. The onset and progression of obesity, diabetes and DN in humans are primarily due to lifestyle factors; thus, their study in humans is challenging. Given the complexity of this process, it is imperative to develop animal models that recapitulate DN pathophysiology in humans. Animal models of DN are expected to exhibit all the features observed in human DN. These include progressive albuminuria, decreased renal function, distinct glomerular histological alterations (glomerular hypotrophy, GBM thickening, mesangial expansion, foot process effacement and nodular sclerosis) and tubulointerstitial lesions (Alicic et al., 2017). Numerous diabetic rodent models have been developed, including streptozotocin-induced type 1 diabetic rodents, *db/db* mice, KK-*Ay* mice and Wistar fatty rats for T2DM (Kitada et al., 2016). Although these diabetic rodent models have been demonstrated to be useful for increasing our understanding of hyperglycemia-induced renal injury, a rodent model that perfectly mimics all the characteristics of human DN has not yet been developed (Allen et al., 2004).

Over the past few decades, zebrafish have become an attractive vertebrate in research. The features of zebrafish, such as low cost, high fecundity, external development, a transparent body until the juvenile stage and easy genome editing, have provided researchers with a well-established model system for embryogenesis, developmental biology and human disease studies (Dooley and Zon, 2000; Zang et al., 2022b). Embryonic and adult zebrafish kidney structures, such as basal functional units (nephrons), are highly conserved among mammals (McCampbell and Wingert, 2014). Zebrafish models have emerged from studies of kidney development, renal regeneration and several types of kidney diseases. Researchers have attempted to develop zebrafish DN models using genetic techniques. For example, leptin (*lepb*)-deficient zebrafish (He et al., 2021) or *Pdx1* knockout zebrafish (Wiggenhauser et al., 2022) develop DN-like phenotypes. However, a zebrafish model that mimics the etiology and pathogenesis of human DN is required.

Zebrafish models of obesity and diabetes have been developed using overnutrition treatments or genetically edited transgenic or mutant lines (Zang et al., 2018). We have previously developed a zebrafish T2DM model using wild-type zebrafish under overnutrition conditions (Zang et al., 2017). Moreover, transgenic zebrafish with insulin-resistant skeletal muscles [zMIR, *Tg(acta1:dnIGF1R-EGFP)*] (Maddison et al., 2015) demonstrate elevated fasting blood glucose (FBG) levels compared to those in wild-type zebrafish, indicating that zMIR treatment with overfeeding generates a robust T2DM model. Furthermore, as albuminuria (or proteinuria) is clinically characterized as an early predictor of DN progression, the next issue was the measurement of proteinuria in zebrafish. Zhou and Hildebrandt (2012) created a transgenic zebrafish model expressing GFP-tagged vitamin D-binding protein [VDBP, encoded by *gc*; *Tg(l-fabp::VDBP-GFP)*] as a tracer for proteinuria, as VDBP in zebrafish is the homolog closest to mammalian albumin. Proteinuria leakage was detected by measuring the GFP fluorescence signal upon podocyte injury, indicating that proteinuria can be monitored in zebrafish. Thus, we sought to answer the following questions: (1) do obesity-induced diabetic zebrafish develop DN as humans do; (2) if yes, is it possible to restore damaged kidney function through calorie restriction (CR), and (3) does this model show the same molecular mechanism as that in human DN?

In the present study, we created a stable double-transgenic line, zMIR/VDBP, by crossing the zMIR and VDBP zebrafish lines. Subsequently, we conducted an overfeeding experiment to induce hyperglycemia, monitored proteinuria, analyzed renal histopathology, assessed the impacts of CR, and conducted RNA sequencing (RNA-seq) and comparative transcriptome analyses to elucidate the underlying molecular mechanisms.

## RESULTS

### Overfed adult zMIR/VDBP zebrafish develop obesity and diabetes with proteinuria

**Fig. 1.**
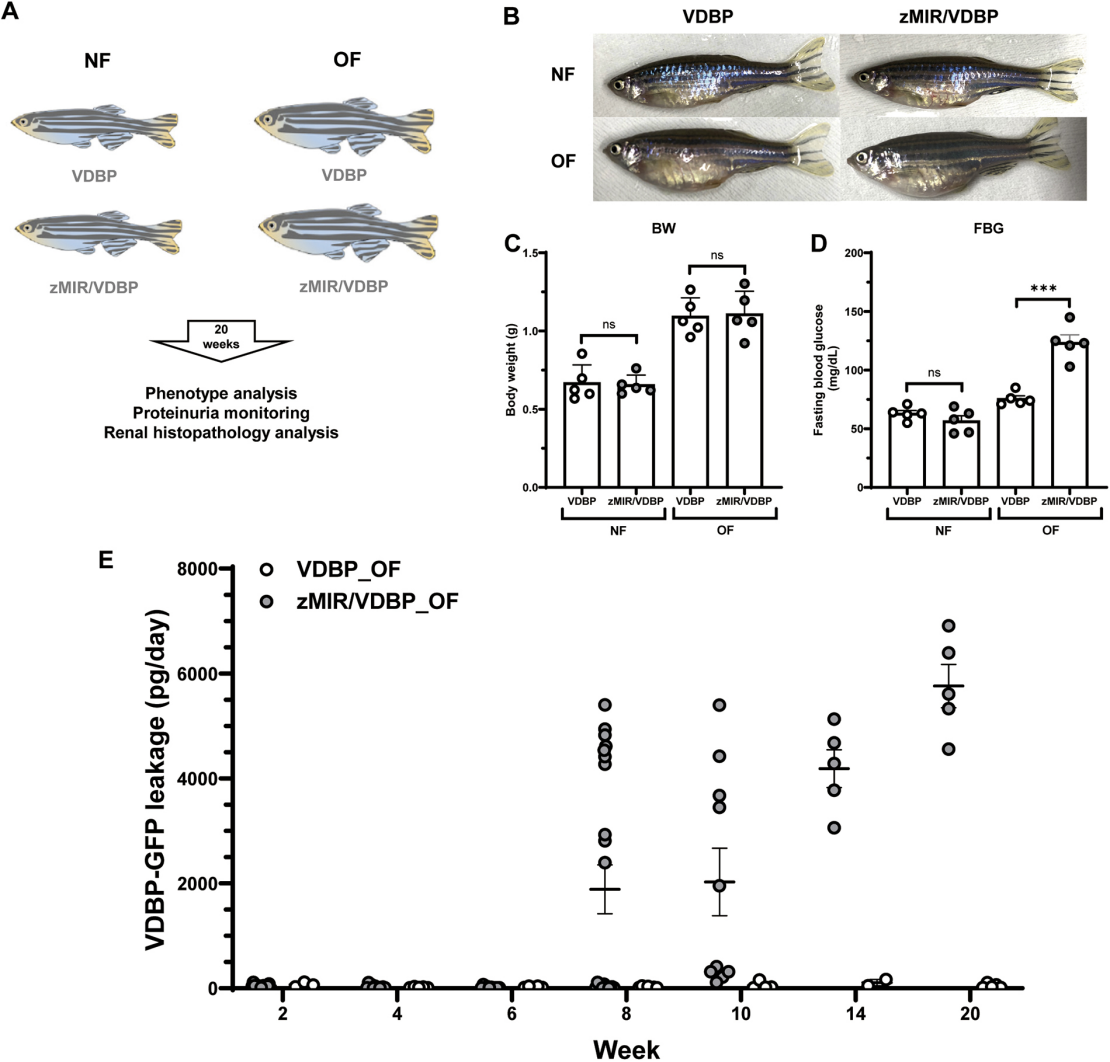
**Overfed zMIR/VDBP zebrafish exhibit obesity, hyperglycemia and glomerular filtration barrier dysfunction.** (A) Experiment schema of overfeeding to induce diabetes. Female VDBP and zMIR/VDBP zebrafish were divided into normal-feeding (NF) and overfeeding (OF) groups. Proteinuria measurements were performed every 2 weeks for a total of 20 weeks. At the endpoint, renal histopathology was analyzed using Hematoxylin and Eosin staining and transmission electron microscopy. (B) Representative images of VDBP and zMIR/VDBP zebrafish with NF and OF for 20 weeks. (C,D) Quantification of body weight (BW) (C) and fasting blood glucose (FBG) (D) of the four groups at the endpoint of the feeding experiment (*n*=5). (E) Proteinuria changes in the urine every 2 weeks during the overfeeding experiment in overfed VDBP and zMIR/VDBP zebrafish (*n*=5-22). The VDBP-GFP signal was measured using ELISA as an indicator of albuminuria. Data are presented as means±s.e.m. ns, not significant; ***P<0.001 versus VDBP with OF; Bonferroni–Dunn multiple comparisons test.

Adult female VDBP and zMIR/VDBP zebrafish were divided into four groups and subjected to normal feeding or overfeeding (Fig. 1A). Proteinuria measurements were performed every 2 weeks for a total duration of 20 weeks. The two groups treated with overfeeding showed large bodies and significantly increased body weights compared to those of the normal feeding groups (Fig. 1B,C). Overfed VDBP fish showed increased FBG levels compared to those of normally fed fish (*P*<0.05). Notably, the overfed zMIR/VDBP fish exhibited the highest FBG levels compared to those of the other three groups (Fig. 1D). These results indicated that zMIR/VDBP overfeeding leads to obesity and T2DM. Accompanied by the induction of overfeeding, proteinuria was first detected in overfed zMIR/VDBP fish in week 8 (1888±466 pg/day, indicated as mean±s.e.m.) and increased dramatically until the end of the observed duration (5750±411 pg/day) (Fig. 1E). Additionally, no proteinuria was detected in the VDBP and zMIR/VDBP zebrafish fed a normal diet during the experiment. Increased proteinuria over time indicated that glomerular filtration barrier dysfunction occurred in overfed zMIR/VDBP zebrafish. In contrast, although proteinuria was also detected in overfed VDBP fish, the amount was comparatively small (<100 pg/day).

### Overfed zMIR/VDBP zebrafish show renal pathological alterations as signs of DN

**Fig. 2.**
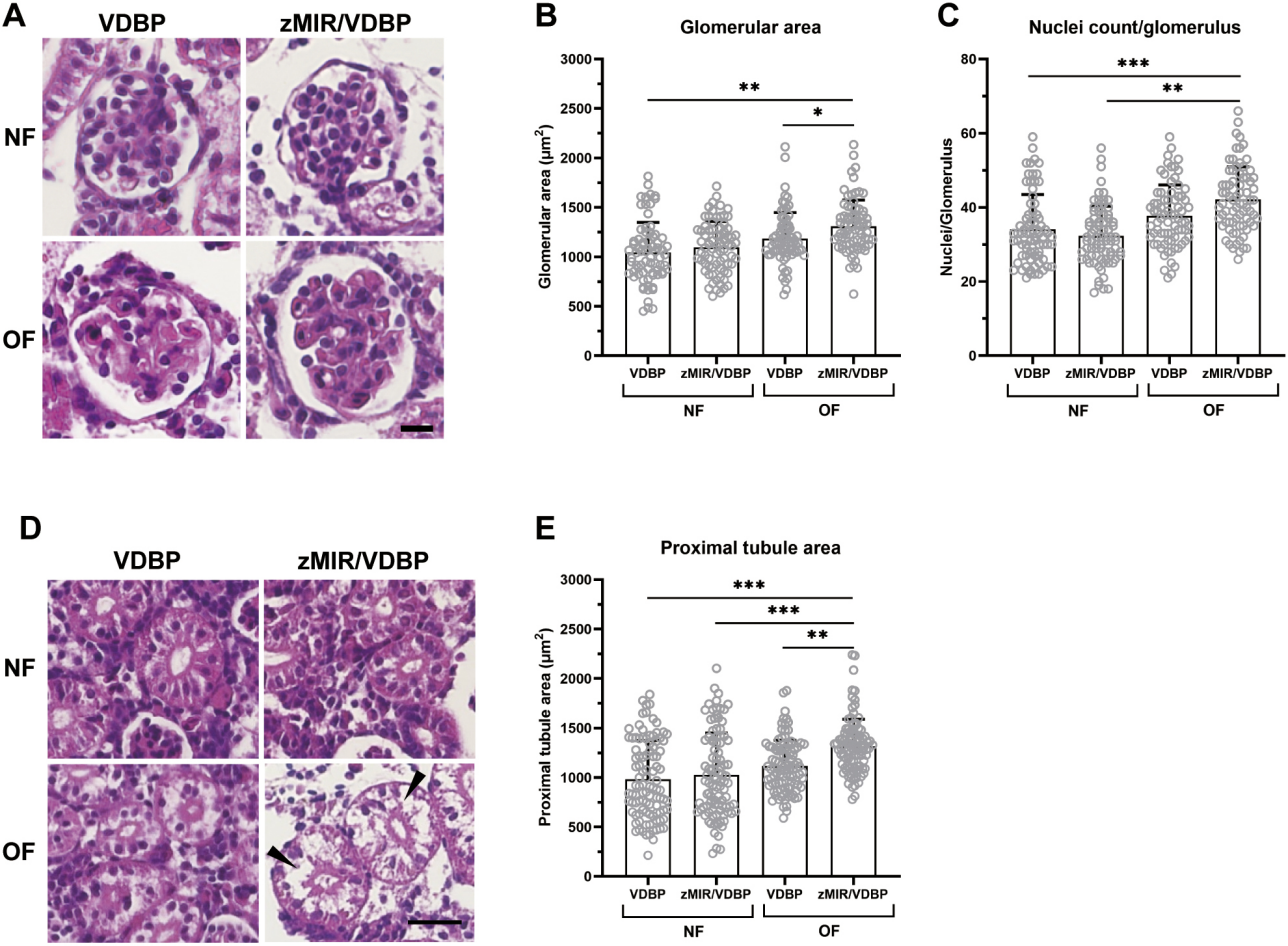
**Overfed zMIR/VDBP zebrafish develop glomerular morphological changes.** (A) Hematoxylin and Eosin (H&E)-stained paraffin kidney sections revealed marked glomerular hypertrophy in overfed zMIR/VDBP zebrafish. Scale bar: 10 μm. (B,C) Quantification of glomerular area (B) and average nuclear count per glomerular cross-section (C) in the four groups. At least 75 glomeruli from five fish per group were measured on three sections per fish. (D) H&E-stained sections from overfed zMIR/VDBP zebrafish showed pale cytoplasms with proximal tubule enlargement. Black arrowheads indicate the cavities in the proximal tubule, which may be caused by ectopic lipid accumulation. Scale bar: 20 μm. (E) The cross-sectional area of the proximal tubules in overfed zMIR/VDBP fish was significantly larger than that in those fed a control diet or overfed VDBP fish. At least 100 tubular cross-sections were measured in each group. Data are presented as mean±s.e.m. *P<0.05; **P<0.01; ***P<0.001; Bonferroni–Dunn multiple comparisons test.

To evaluate glomerular phenotypes in zMIR/VDBP fish, we investigated the renal histopathology of the four treatment groups. The mean glomerular area of overfed zMIR/VDBP fish was significantly larger than that of the normally fed groups (*P*<0.05), whereas no significant difference was observed for overfed VDBP fish (Fig. 2A,B). The overfed zMIR/VDBP fish showed a significantly higher average number of nuclei per glomerulus compared to that for the other three groups (Fig. 2C). In the proximal tubules, normally fed fish displayed regular cell spacing and eosinophilic cytoplasm, whereas overfed VDBP and zMIR/VDBP fish exhibited a pale cytoplasm and irregular cell spacing (Fig. 2D). Moreover, cavities were observed in the enlarged proximal tubules of overfed zMIR/VDBP mice (black arrowheads), which may have been caused by ectopic lipid accumulation. The proximal tubule area was significantly increased, indicating that proximal tubule enlargement occurred in the overfed zMIR/VDBP fish (*P*<0.01; Fig. 2E).

**Fig. 3.**
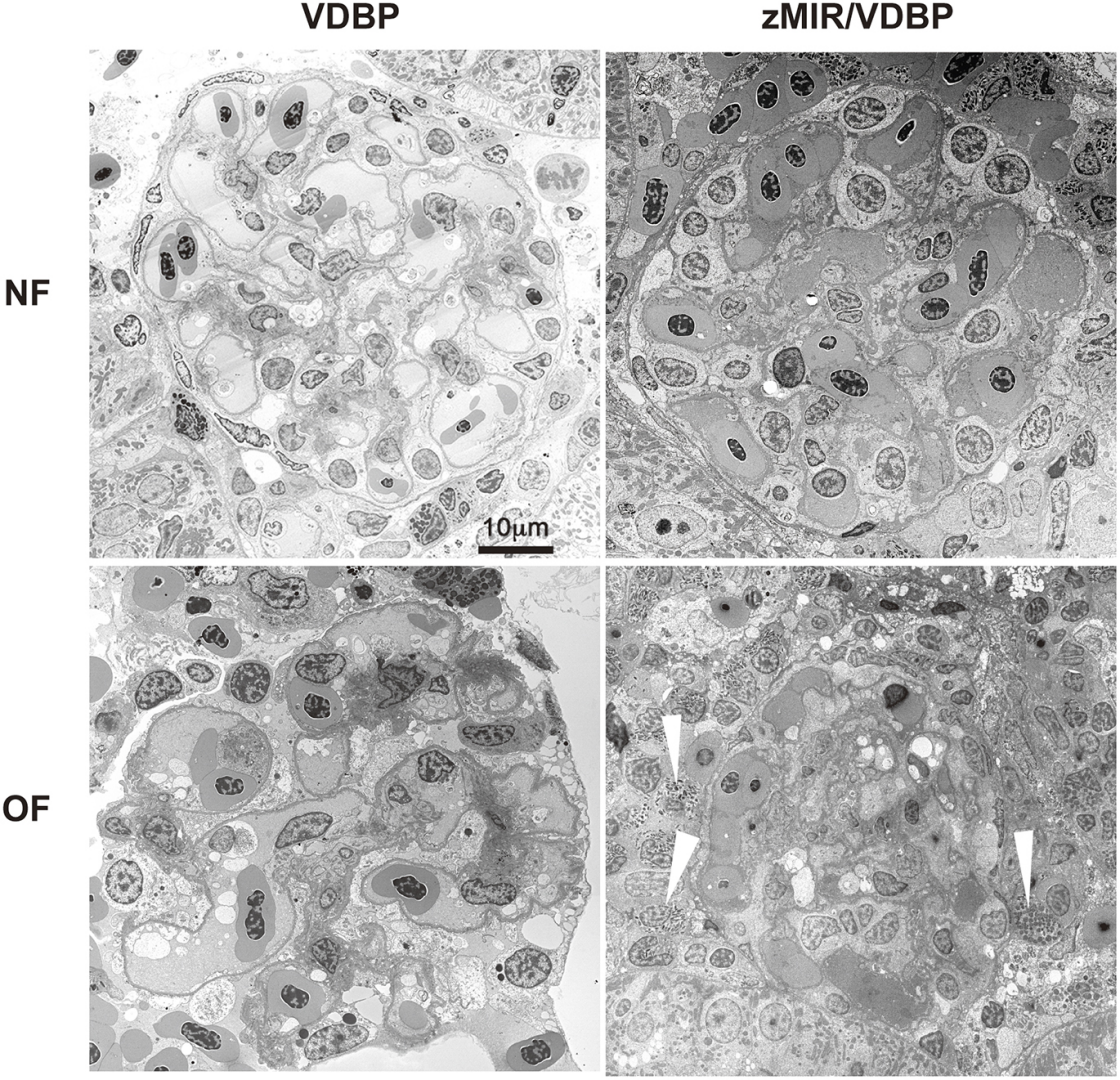
**Transmission electron microscopy images of glomeruli from the four groups of zebrafish.** White arrowheads show eosinophil infiltration in overfed zMIR/VDBP fish. Images are representative of four fish per group. Scale bar: 10 μm.

**Fig. 4.**
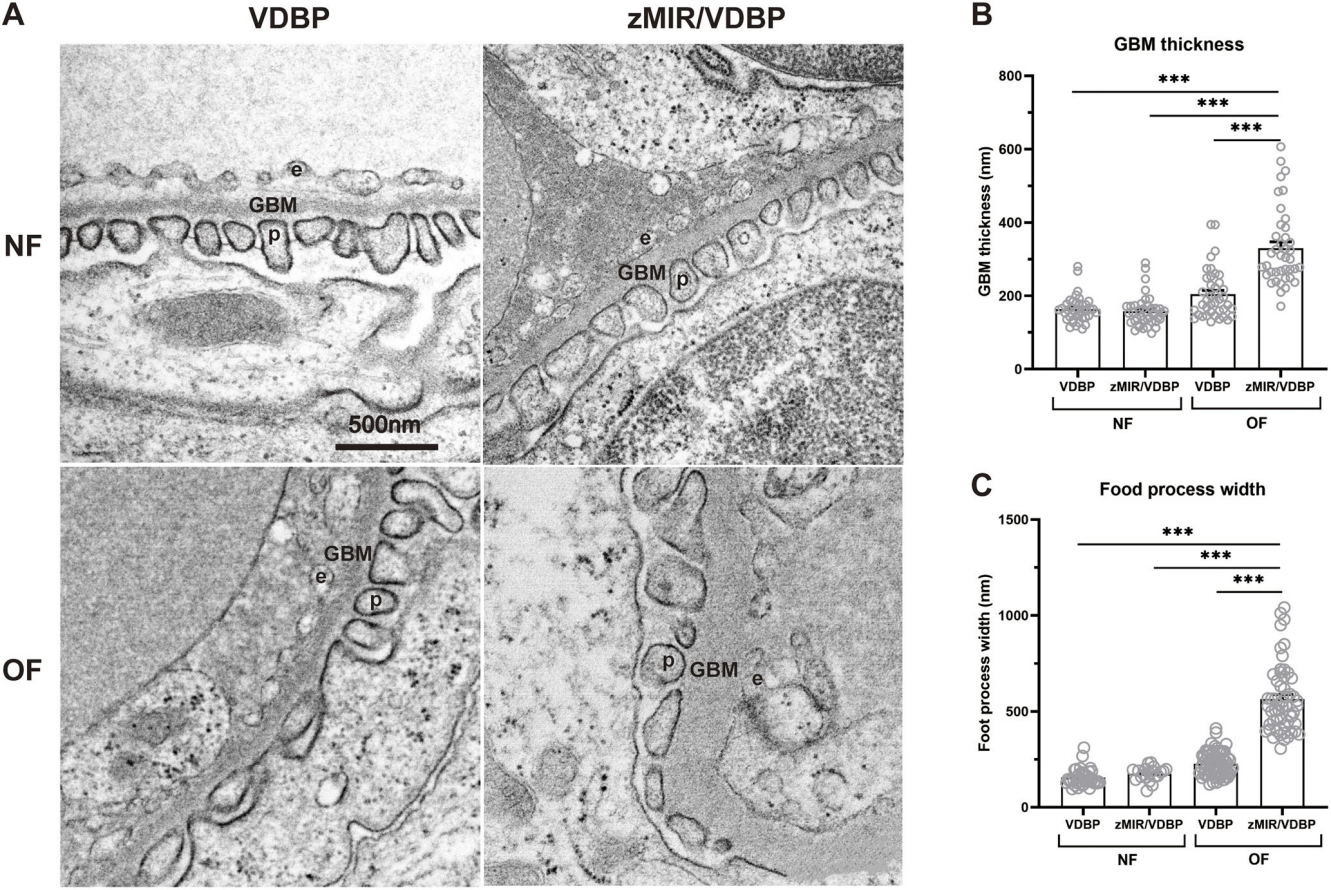
**Overfed zMIR/VDBP zebrafish develop thick glomerular basement membranes.** (A) Representative transmission electron microscopy images of the glomerular basement membrane (GBM) and foot processes of the four groups. The podocytes (p), endothelium (e) and GBM are marked on the images. Scale bar: 500 nm. (B) Summary of the GBM thickness of the four groups of zebrafish. GBM thickness was measured at ten randomly assigned points in each image from four fish/group. (C) Foot process width analysis revealed that foot process effacement was present in overfed zMIR/VDBP fish. At least 20-70 podocyte foot processes were measured in each group. Data are presented as mean±s.e.m. ***P<0.001; Bonferroni–Dunn multiple comparisons test.

Transmission electron microscopy (TEM) analysis revealed that normally fed VDBP and zMIR/VDBP fish showed normal glomerular structures, whereas overfed fish exhibited progressive glomerular destruction, including eosinophil infiltration (white arrowheads) (Fig. 3). High-magnification images showed endothelial cell sclerosis, mesangial interposition, mesangial expansion, podocyte foot process effacement, eosinophil infiltration and even glomerular sclerosis in the overfed zMIR/VDBP fish (Fig. S1). Thickening of the GBM is another primary sign of DN. We found a significant increase in GBM thickness in overfed zMIR/VDBP fish compared to that in overfed VDBP fish (*P*<0.001; Fig. 4A,B). Overfed zMIR/VDBP fish exhibited focally disorganized and clumped podocyte foot processes. The qualitative assessment indicated obvious effacement of the podocyte foot process (Fig. 4C). These data indicate that overfed zMIR/VDBP fish developed pathological findings similar to those of humans, leading us to conclude that this was a novel zebrafish DN model. Hereafter, we refer to overfed zMIR/VDBP fish as zebrafish with DN or DN fish.

### CR alleviates glomerular damage in zebrafish with DN

**Fig. 5.**
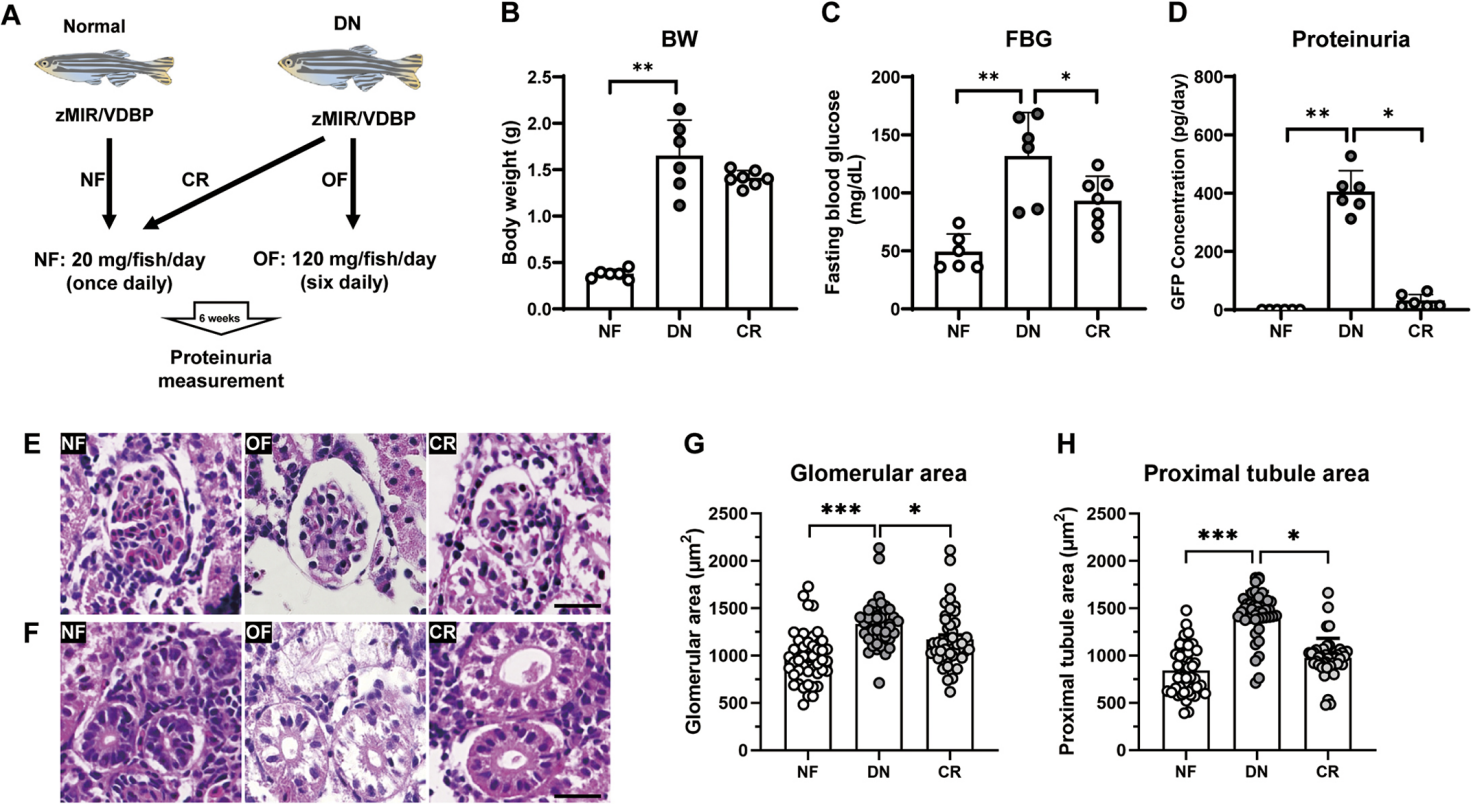
**Calorie restriction restores the glomerular filtration barrier dysfunction in overfed zMIR/VDBP zebrafish.** (A) Experiment schema. Zebrafish with DN were divided into calorie restriction [CR, fed with the same amount as normally fed (NF) fish] and continuously overfeeding (DN) groups. The experiment duration was 6 weeks. Proteinuria measurement was performed at the endpoint, and the renal histopathology was analyzed by H&E staining. (B) CR caused a slight decrease in body weight (BW) compared to that in DN fish (*n*=6-7). (C) CR caused a significant decrease in fasting blood glucose (FBG) levels compared to those in DN fish (*n*=6-7). (D) CR treatment significantly decreased proteinuria leakage compared to that in zebrafish with DN (*n*=6-7). (E) Representative images of the glomeruli of the three groups of zebrafish. CR treatment significantly decreased the glomerular hypertrophy observed in zebrafish with DN. Scale bar: 20 μm. (F) Representative images of the proximal tubule area of the three groups of zebrafish. Scale bar: 20 μm. (G,H) CR treatment resulted in a return to normal glomerular area (G) and proximal tubule size (H). Each group had 45 separate glomeruli and proximal tubules analyzed from three fish in each group. Data are presented as mean±s.e.m. *P<0.05; **P<0.01; ***P<0.001; Bonferroni–Dunn multiple comparisons test.

It is well known that CR can protect against acute kidney injury in humans (Johnsen et al., 2020), and zebrafish have been reported to have a high capacity for the regeneration of various organs. We hypothesized that CR may reverse kidney function in zebrafish with DN. Zebrafish with DN were randomly assigned to the overfeeding and CR groups, with the CR group fed the same amount as the normally fed fish for 6 weeks (Fig. 5A). CR caused slight weight loss compared to the weights of DN fish, with no significant difference (Fig. 5B). However, FBG levels in CR fish were significantly lower than those in DN fish (*P*<0.05; Fig. 5C). Furthermore, the amount of VDBP-GFP protein leakage (proteinuria) significantly decreased after CR treatment compared to that in DN fish (394±185 versus 21±19 pg/day, *P*<0.05; Fig. 5D). Histological analysis demonstrated that CR treatment led to a restoration of normal glomerular area (Fig. 5E,G) and proximal tubule size (Fig. 5F,H). Therefore, we concluded that CR could improve kidney function in the zebrafish DN model.

### Altered gene expression profiling in the kidney of the zebrafish DN model

To identify the gene factors and related signaling pathways during DN progression in zebrafish, we conducted RNA-seq transcriptomic analysis using whole-kidney tissues from normally fed (control) and overfed zebrafish with DN. We identified 582 upregulated and 969 downregulated differentially expressed genes (DEGs) in DN compared to control zebrafish. The results of Gene Ontology (GO) analysis using these DEGs are shown in Fig. S2. We performed ClueGO analysis (Bindea et al., 2009) to visualize gene cluster interactions in a functionally grouped network using GO enrichment maps. Upregulated DEGs were associated with anatomical structure morphogenesis, signaling regulation, integrin-mediated cell adhesion and actin cytoskeleton organization (Fig. S3A). Downregulated DEGs were associated with the regulation of mitotic nuclear division, non-coding RNA metabolic processes, DNA metabolic processes and nucleic acid metabolic processes (Fig. S3B).

KEGG pathway analysis (Kanehisa et al., 2011) showed that the upregulated DEGs were significantly enriched in ‘focal adhesions’, ‘extracellular matrix interactions’ and the proto-oncogenic ‘phosphatidylinositol 3 kinase (PI3K)/protein kinase B (Akt) signaling pathway’ (Fig. S4A). Downregulated DEGs were enriched in ‘cell cycle’, ‘Fanconi anemia pathway’, ‘vitamin digestion and absorption’, ‘TGF-β signaling pathway’ and ‘calcium signaling pathway’ (Fig. S4B).

### Pathways involved in kidney damage in DN zebrafish

To identify the key signaling pathways and gene networks in zebrafish with DN, we performed Ingenuity Pathway Analysis (IPA) for further functional annotation and prediction. Canonical pathway analysis, which determined the altered established metabolic and cell signaling pathways, identified 12 significantly regulated pathways in zebrafish with DN (z-score>2; Fig. S4C). The phosphatase and tensin homolog (PTEN) signaling pathways (Fig. S5) and pathways involved in cell cycle control of the chromosomal replication were negatively regulated, whereas other pathways were activated in zebrafish with DN, such as the PI3K/Akt or FAT10 cancer signaling pathway. Next, the association between the DEGs in zebrafish with DN and toxicity was determined by IPA. When setting the display category to ‘nephrotoxicity’, nine toxicity functions were identified [−log(*P*-value)>1.3, z-score>2], including ‘renal proliferation’, ‘renal damage’, ‘glomerular injury’, ‘renal thrombosis’, ‘renal necrosis/cell death’, ‘renal inflammation’, ‘renal nephritis’, ‘nephrosis’ and ‘renal fibrosis’ (Fig. S4D). Fig. S4E shows the activated ‘apoptosis of renal tubule’ network (‘renal necrosis/cell death’ category) with details of the related DEGs and the relationships between the DEGs and this function. In the ‘renal damage’ category, ‘reperfusion injury of kidney’ networks were also activated in zebrafish with DN (Fig. S4F).

### Comparative transcriptome analysis reveals common pathophysiological pathways in zebrafish and human DN

**Fig. 6.**
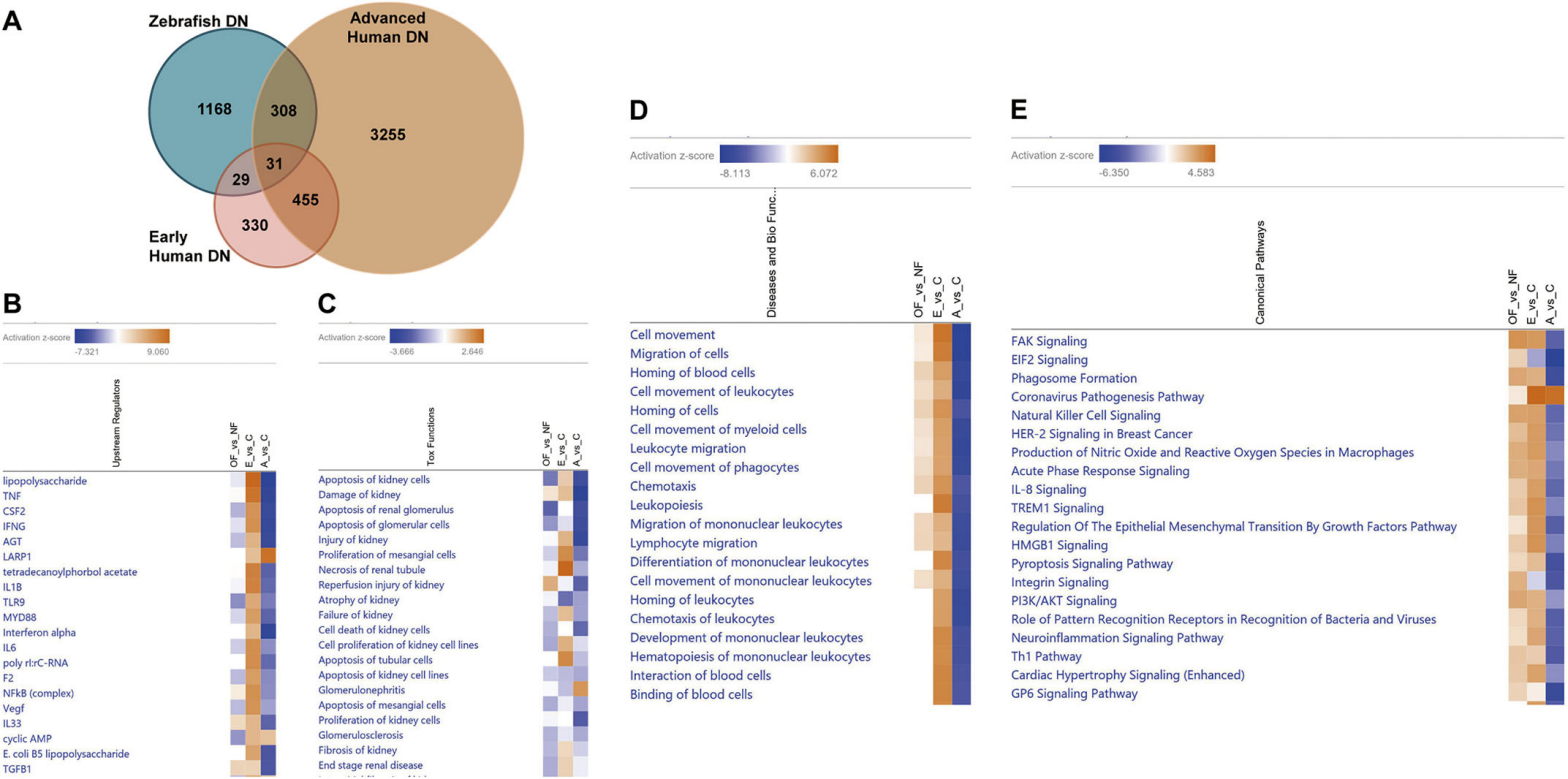
**Comparative transcriptome analysis results for zebrafish DN, early human DN and advanced human DN.** (A) Venn diagram for the overlapping differentially expressed genes (DEGs) among zebrafish DN, early human DN and advanced human DN. (B-E) Heatmaps show the results of comparing functional Ingenuity Pathway Analysis (IPA) of the three groups, including the top 20 upstream regulators (B), toxicity functions (nephrotoxicity) (C), disease and biofunctions (D) and canonical pathways (E). Regulators, functions and pathways are ranked based on the z-score that predicts activation (orange) and inhibition (blue). OF_vs_NF, overfeeding versus normal feeding; E_vs_C, early human DN versus human control; A_vs_C, advanced human DN versus human control.

**Fig. 7.**
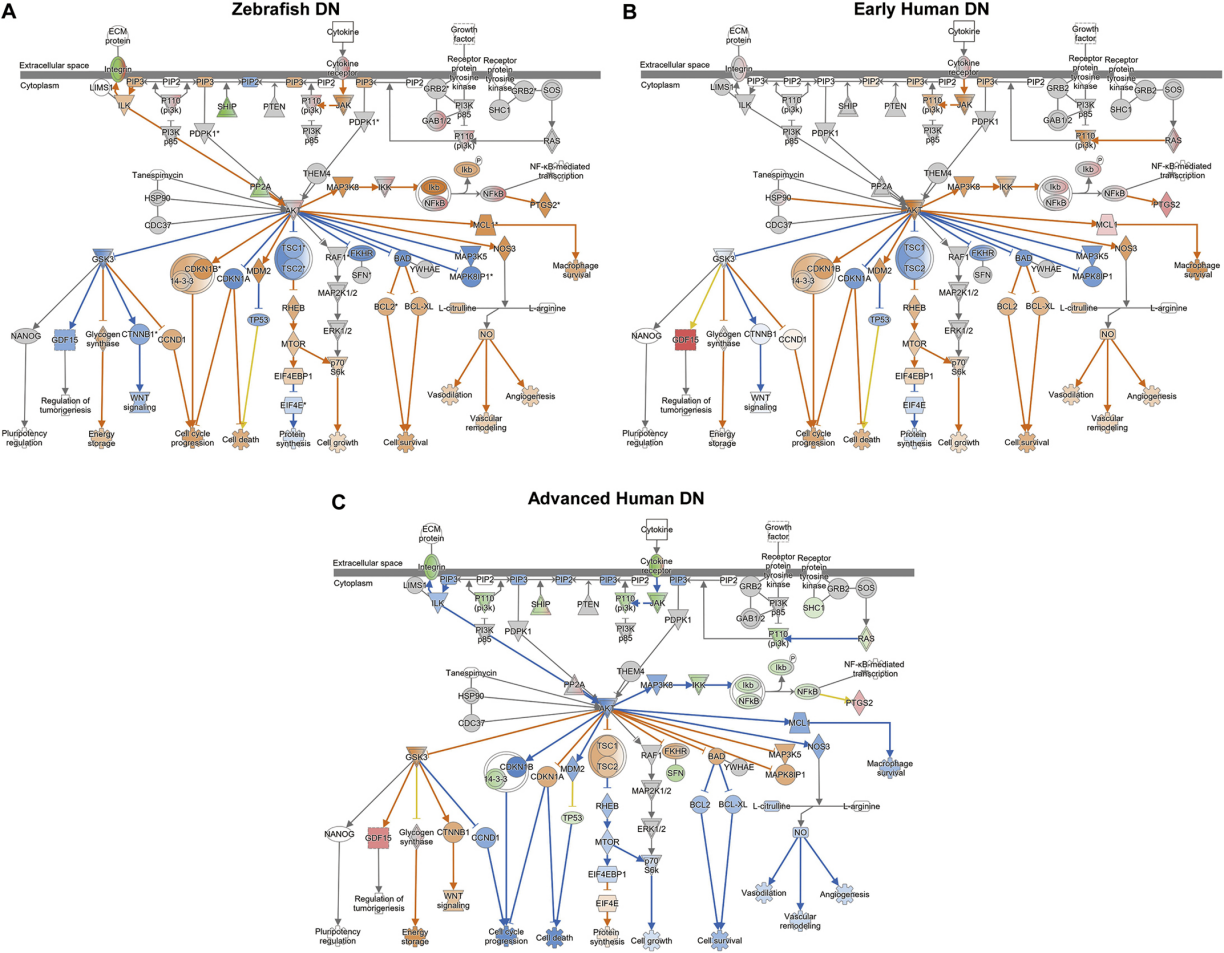
**PI3K/Akt signaling pathways identified by IPA in the zebrafish DN, early human DN and advanced human DN.** PI3K/Akt signaling pathway was activated in zebrafish DN (A) and early human DN (B) but was inhibited in advanced DN (C). Upregulated and downregulated DEGs are shown in red and green, respectively, and the predicted activation and inhibition effects of signaling intermediates and pathways are shown as orange and blue lines, respectively. Asterisks in A indicate that multiple identifiers in the dataset file map to a single gene or protein in the global molecular network.

To investigate whether zebrafish with DN share common transcriptomic pathways with humans with DN, we conducted a comparative transcriptome analysis of zebrafish and humans with DN. The RNA-seq dataset GSE142025 was obtained from the Gene Expression Omnibus and served as a reference human DN gene expression profile. This dataset was derived from human kidney biopsy samples from the unaffected portion of tumor nephrectomies categorized into normal kidney function; control, early DN (estimated glomerular filtration rate >90 ml/min/1.73 m^2^ and urinary albumin-to-creatinine ratio <300 mg/g), and advanced DN (estimated glomerular filtration rate <90 ml/min/1.73 m^2^ and urinary albumin-to-creatinine ratio >300 mg/g) (Fan et al., 2019). There were 60 overlapping DEGs between zebrafish DN and early human DN, whereas 339 overlapping DEGs were identified between zebrafish DN and advanced human DN (Fig. 6A). Only 31 DEGs were identified in all three datasets. Next, we performed comparative analyses of the three DEG datasets using IPA software. Upstream regulator analysis aims to identify a cascade of upstream transcriptional regulators that can explain the observed changes in gene expression. The three most common upstream regulators across the three datasets were lipopolysaccharide, TNF and CSF2 (Fig. 6B). Additionally, the top three common toxicity functions specific to nephrotoxicity were ‘apoptosis of kidney cells’, ‘kidney damage’ and ‘apoptosis of renal glomeruli’ (Fig. 6C). Disease and biofunction analyses revealed that the most commonly regulated functions were ‘cell movement’, ‘migration of cells’ and ‘homing of blood cells’ (Fig. 6D). The common top three canonical pathways were ‘FAK signaling’, ‘EIF2 signaling’ and ‘phagosome formation’ (Fig. 6E). Interestingly, the predicted activation state showed high similarities between zebrafish DN and advanced human DN in the upstream regulator and nephrotoxicity function analyses. In contrast, disease, biological function and canonical pathway analyses showed a higher similarity between zebrafish DN and early human DN than between zebrafish DN and advanced human DN. For example, the top identified toxicity function, ‘kidney cell apoptosis’, was inhibited in zebrafish DN and advanced human DN, whereas it was activated in early human DN (Fig. S6). In addition, the top-regulated canonical signaling pathway, ‘FAK signaling’, was activated in zebrafish DN and early human DN but inhibited in advanced human DN (Fig. S7). The PI3K/Akt signaling pathway, which has been demonstrated to play an important role in DN progression, exhibited similar activation states as FAK signaling (Fig. 7A-C).

### Metformin ameliorates the proteinuria leakage and FBG levels in the DN zebrafish model

**Fig. 8.**
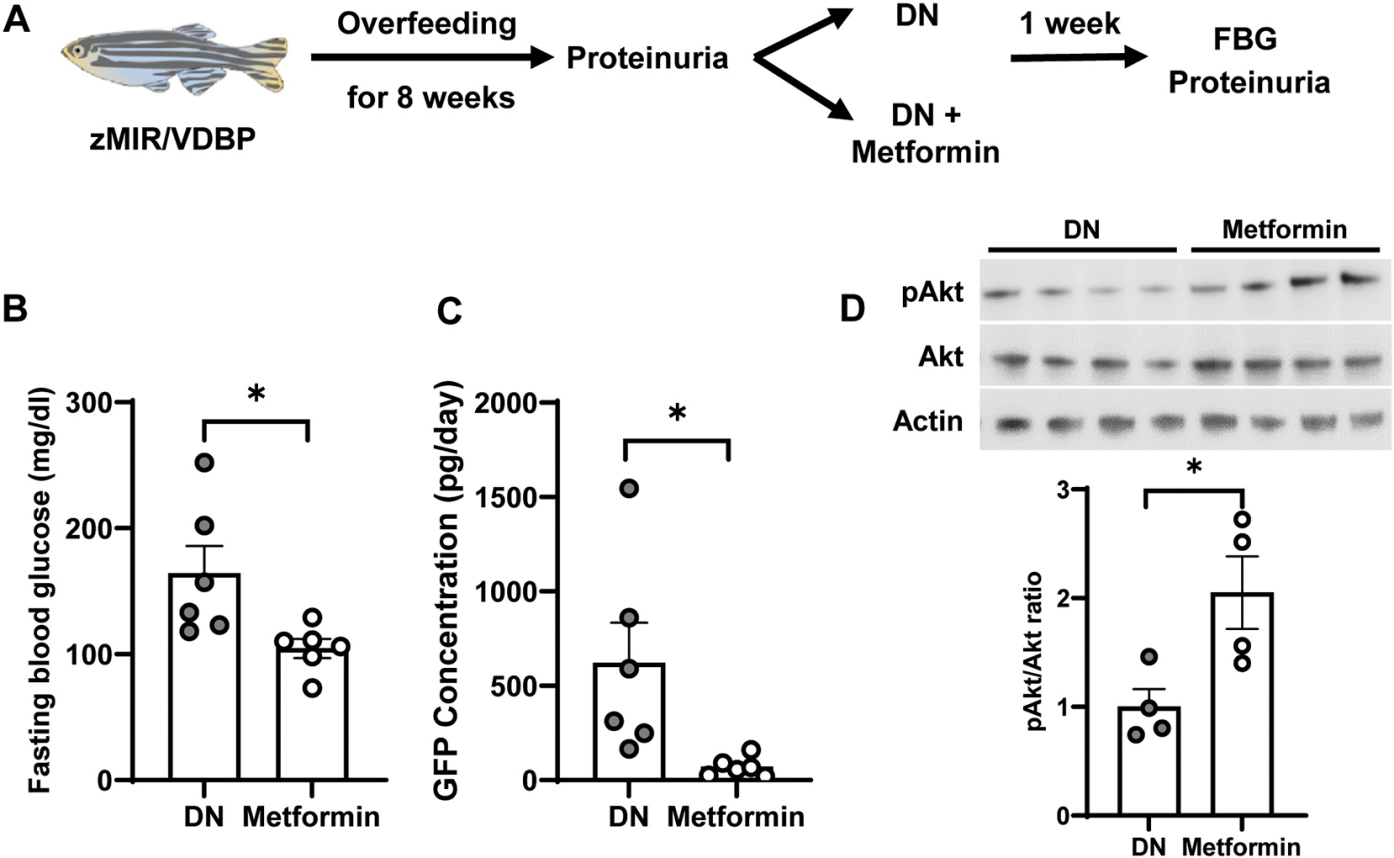
**Metformin ameliorates the proteinuria leakage and FBG levels in the DN zebrafish model.** (A) Schematic of overfeeding and metformin treatment. zMIR/VDBP zebrafish were overfed for 8 weeks and proteinuria was measured for DN fish selection. Then, DN fish were divided into two groups of DN only and DN with metformin treatment, and continued overfeeding for 1 week. (B) Metformin caused a significant decrease in fasting blood glucose (FBG) levels compared to those in DN fish (*n*=6). (C) Metformin treatment significantly decreased proteinuria leakage (*n*=6). (D) The ratio of phosphorylated Akt (pAkt)/total Akt expression in kidney tissue was higher in metformin-treated DN zebrafish than in the control DN group (*n*=4). Data are presented as mean±s.e.m. *P<0.05; two-tailed unpaired *t*-test.

Metformin protects renal cells under hyperglycemic conditions by modulating the PI3K/Akt signaling pathway (Ravindran et al., 2017). To confirm the role of the PI3K/Akt signaling pathway in DN progression in zebrafish, we treated DN zebrafish with metformin for 7 days (Fig. 8A). After metformin treatment, FBG levels were significantly lower in metformin-treated DN zebrafish than in untreated DN zebrafish (Fig. 8B). Furthermore, DN zebrafish showed a significant suppression of proteinuria leakage from 621±213 pg/day (before) to 70±213 pg/day (after) (Fig. 8C). Western blot analysis revealed that metformin-treated DN zebrafish showed significantly higher Akt activity than that in untreated DN fish (*P*<0.05, Fig. 8D).

## DISCUSSION

A novel zebrafish DN model, zMIR/VDBP, which develops from obesity to T2DM, and finally to DN, was created. This model develops features similar to those of early and advanced human diabetic kidney disease, including persistent albuminuria, glomerular hypertrophy, proximal tubule enlargement, GBM thickening, foot process effacement and glomerular sclerosis. This is the first report to monitor proteinuria in zebrafish and develop DN similar to the progression and pathogenesis of human DN. Moreover, RNA-seq revealed that zebrafish with DN share common pathological transcriptome pathways with human patients with DN. These findings suggest that the zebrafish DN model can be used to identify putative pharmacological targets and develop novel therapies for human DN.

Two studies have reported kidney dysfunction in zebrafish, which can be used as a DN model. He et al. (2021) generated a *lepb*-deficient zebrafish line (*lepb^−/−^*) and found that adult *lepb^−/−^* fish exhibited T2DM features, glomerular hypertrophy and GBM thickening as early signs of DN. However, they did not observe mesangial expansion, podocyte damage or tubular cell damage in the kidney tissues of *lepb^−/−^* zebrafish, indicating that the fish developed mild renal injury. Another mutant line, *pdx1^−/−^*, was recently established as a zebrafish DN model (Wiggenhauser et al., 2022). Larvae and adult *pdx1^−/−^* mutants exhibit signs of early diabetic kidney disease, such as glomerular hypertrophy and GBM thickening. Although vascular endothelial growth factor (VEGF) signaling was downregulated in *pdx1^−/−^* mutants, no morphological microvascular phenotypes were observed, indicating that *pdx1^−/−^* mutants are a model for early diabetic kidney disease. Compared to these two mutant models, our zebrafish DN model exhibited not only three particular histopathological characteristics of DN – glomerular hypertrophy, mesangial expansion and GBM thickening (Tervaert et al., 2010) – but also human-like DN end-stage symptoms, such as massive proteinuria and glomerular sclerosis (Alicic et al., 2017). In addition, a notable study evaluated the effects of a high-calorie diet on adult wild-type zebrafish (Zeitler et al., 2022). As a result, fish fed a high-calorie diet developed glomerular filtration barrier dysfunction and podocyte injury without elevated FBG levels. Thus, this model should be used as an obesity-related kidney disease model rather than as a DN model. Our DN model showed continuous hyperglycemia as an initiation factor and obesity as a progression factor, thus gaining an advantage as a human DN model. Another advantage of this DN model is that it enables monitoring of proteinuria in adult zebrafish. In a previous study, researchers injected fluorescent 70 kDa dextran mimicking albumin to examine renal filtration function in zebrafish larvae (Wiggenhauser et al., 2022). In the present study, we used the characteristics of transgenic fish to evaluate proteinuria in adult fish. Our DN model resolved this problem in a non-invasive manner, and proteinuria was observed over time. Collectively, these findings indicate that this model enables the elucidation of the pathogenic mechanisms involved in the initiation and progression of human diabetic kidney diseases.

In addition to the nephrotic phenotypes resulting from hyperglycemia, the zebrafish model showed molecular mechanisms similar to those of human DN. Wang et al. (2022) analyzed a single-cell RNA-seq dataset (GSE131882) from the kidney tissues of human DN samples and unaffected samples. They identified the top three GO enrichment terms among the upregulated and downregulated DEGs. The top GO enrichment terms, namely, ‘cell adhesion’, ‘plasma membrane’ and ‘apical plasma membrane’, were also found in zebrafish with DN (Fig. S2). Cell adhesion involves the differentiation, activation and communication between immune cells (Howell and de Leeuw, 2018; Le Saux et al., 2020). The levels of adhesion molecules and cytokines change in DN and are essential mediators of glomerular injury, highlighting their diagnostic role in renal microvascular complications (Wada and Makino, 2013; Zhang et al., 2020). Additionally, KEGG pathway analysis in humans revealed that the upregulated DEGs were mainly enriched in the focal adhesion and PI3K/Akt signaling pathways, whereas the downregulated DEGs were enriched primarily in the calcium signaling pathway. Similar results were observed for the functionality of the DEGs based on the zebrafish DN RNA-seq data (Fig. S4A).

PTEN is a protein and lipid phosphatase that plays essential roles in apoptosis, cell cycle arrest and cell migration (Chu and Tarnawski, 2004). It is also a potent tumor suppressor that antagonizes PI3K/Akt signaling and the downstream mammalian target of the rapamycin (mTOR) signaling pathway (Miranda-Gonçalves et al., 2018). PTEN plays a significant role in DN by regulating renal tubulointerstitial fibrosis and the epithelial-mesenchymal transition via the PI3K/Akt/mTOR signaling pathway (Khokhar et al., 2020). As a result of hyperglycemic stimulation of PI3K and inhibition of PTEN, the Akt/mTOR pathway is activated, leading to the development of DN through abnormal podocyte autophagy, renal fibrosis, enlargement of the mesangial matrix and thickening of the basement membrane (Estacio and Schrier, 2001). Hence, the PI3K/Akt signaling pathway is deemed a promising therapeutic target for modulating autophagy in podocytes and decelerating DN progression. Various reagents have been reported to mitigate renal damage by inhibiting the PI3K/Akt signaling pathway in DN rat models (Chen et al., 2021; Jin et al., 2022; Ou et al., 2021). The present study unveiled that, among the top ten regulated canonical pathways, PTEN signaling was significantly inhibited and the PI3K/Akt/mTOR signaling pathway was activated in our zebrafish DN model (Fig. S4C; Fig. 7A).

To obtain pharmacological evidence for the underlying mechanisms, we treated DN zebrafish with metformin, a well-known anti-diabetic agent. Metformin has been reported to exert nephroprotective effects via multiple signaling pathways, such as activation of the AMP-activated kinase (AMPK)/mTOR pathway or AMPK-independent hypoxia- and lipotoxicity-protective pathways (Ravindran et al., 2017). In this study, a 1-week treatment with metformin drastically improved FBG levels and proteinuria, concomitant with an upregulated phosphorylated Akt (pAkt)/total Akt ratio (Fig. 8). Phosphorylated Akt is involved in glucose intake and glycogen synthesis and may improve insulin resistance (Hajduch et al., 2001). An early DN mouse model showed decreased phosphorylation of Akt in the glomeruli, which caused podocyte cell death (Tejada et al., 2008). Additionally, a previous study reported that metformin ameliorated apoptosis in high-glucose-treated human podocytes by increasing Akt protein expression (Langer et al., 2016). We hypothesize that metformin treatment attenuated proteinuria in our DN zebrafish model by reducing podocyte apoptosis. However, further studies are required to confirm this hypothesis.

The progression of human DN is categorized into the following stages based on urinary albumin excretion: normoalbuminuria, microalbuminuria and macroalbuminuria (Zelmanovitz et al., 2009). To determine the appropriate DN stage for our zebrafish model, we performed a comparative transcriptome analysis of zebrafish DN, early human DN (microalbuminuria) and advanced human DN (macroalbuminuria). Due to the phenotypes of zebrafish with DN, such as high levels of albuminuria (approximately 6 ng/day after 20 weeks of overfeeding) and severe loss of kidney function (glomerulopathy and even glomerular sclerosis), we hypothesized that our zebrafish DN model would be equivalent to that of advanced human DN. The predicted ‘upstream regulators’ and ‘nephrotoxicity’ in zebrafish with DN and advanced human DN showed similar regulation, supporting our hypothesis. However, ‘disease and biofunctions’ and ‘canonical pathways’ analysis showed higher similarity between zebrafish DN and early human DN instead of between zebrafish DN and advanced DN. For example, the PI3K/Akt signaling pathway is activated in zebrafish DN and early human DN but inhibited in advanced human DN. These findings may be attributed to the regenerative and proliferative abilities of zebrafish podocytes (Huang et al., 2013), which may attempt to repair kidney injury, as observed in early human DN. However, further studies are needed to confirm this hypothesis.

In conclusion, we developed a novel zebrafish DN model. Overfed adult zMIR/VDBP zebrafish exhibited a continuous increase in GFP-tagged proteinuria over time. The renal histopathology showed pathological similarities with human DN, including foot process effacement and glomerular sclerosis. Zebrafish DNs share common transcriptional pathways with human pathologies, suggesting that this model serves as a valuable tool for understanding the pathogenesis mechanisms and drug discovery in human DN. In addition, glomerular filtration barrier dysfunction could be restored by caloric restriction and improvement of hyperglycemia (which is likely attributable to the powerful regenerative ability of zebrafish). In the future, this model could be used to explore therapeutic genes in human DN. One limitation of this study was the use of female zebrafish. Although we believe that female zebrafish can easily develop obesity and type 2 diabetes, further studies are needed to test the possibility of developing a DN model using male zebrafish.

## MATERIALS AND METHODS

### Ethics statement

Animal handling procedures were performed according to Japan's Act on Welfare and Management of Animals (Ministry of the Environment of Japan) and complied with international guidelines.

### Zebrafish husbandry and transgenic lines

The transgenic line *Tg(l-fabp::VDBP-GFP)* (Zhou and Hildebrandt, 2012) and skeletal muscle insulin-resistant zebrafish [zMIR; *Tg(acta1:dnIGF1R-EGFP)*] (Maddison et al., 2015) were raised and maintained as previously described (Zang et al., 2021, 2022a) under standard guidelines (Westerfield, 2007). Embryos were collected after the natural spawn, incubated in 0.3× Danieau's solution [17.4 mM NaCl, 0.21 mM KCl, 0.12 mM MgSO_4_, 0.18 mM Ca(NO_3_)_2_ and 1.5 mM HEPES; pH 7.6], and staged as hours post fertilization. EGFP signaling was observed, and images were captured using a BZ-X710 fluorescence microscope (GFP filter; Keyence, Tokyo, Japan). The zebrafish were fed GEMMA Micro 75, 150 or 300 (Skretting, Fontaine-les-Vervins, France) according to their developmental stage or body length.

### Generation of the zMIR/VDBP line

Double-transgenic zMIR/VDBP zebrafish were generated by crossing the *Tg(l-fabp::VDBP-GFP)* and *Tg(acta1:dnIGF1R-EGFP)* lines. Embryos were selected for subsequent breeding based on the following two conditions: (1) specific signal in the muscle and jaw at 5 days post fertilization for zMIR and (2) a specific signal in the vasculature showing overall blurred light at 7 days post fertilization for VDBP (Fig. S8). Larvae with both zMIR and VDBP signals were raised to adulthood and crossed. The offspring were selected according to the conditions described above. Over six generations were raised until 100% of the offspring exhibited both signals.

### Overfeeding experiment

Obesity and T2DM were induced by overfeeding with Otohime B2 (Marubeni Nisshin Feed, Tokyo, Japan), as previously reported (Zang et al., 2017). After 20 weeks of overfeeding, zebrafish were euthanized in an ice-water bath. The entire kidney was dissected and its weight was measured using a normal electronic balance.

### Proteinuria measurement

Adult VDBP or zMIR/VDBP zebrafish were placed in a deep cup containing 50 ml water for 24 h in the dark. 3 ml of system water were collected from each cup and further concentrated to 50 μl using the Amicon Ultra-0.5 Centrifugal Filter Unit (Millipore). The amount of GFP leakage from each fish was measured using a fluorescent GFP enzyme-linked immunosorbent assay (ELISA) kit (ab229403; Abcam) according to the manufacturer's protocol. Fluorescence intensities were measured using a microplate fluorometer (Arvo X2; PerkinElmer Japan). During the overfeeding experiment, proteinuria was measured every 2 weeks. Each treatment group and ELISA were performed in duplicate.

### FBG measurement

Fish were fasted overnight and blood samples were collected from the dorsal aorta using a glass capillary needle (Zang et al., 2015). FBG levels were measured using a handheld blood glucometer (Glutest Ai; Sanwa Kagaku Kenkyusho, Nagoya, Japan).

### Histological staining and TEM

Zebrafish were euthanized and subjected to laparotomy. Zebrafish mesonephros were harvested from three to five fish from each group and fixed with a 4% paraformaldehyde solution for 24 h. To evaluate the light microscopic appearance, tissues were embedded in paraffin and sectioned into 3-μm-thick consecutive sections using a rotary microtome (HM325, Microm International, Germany). Subsequently, these sections underwent staining with Hematoxylin and Eosin (H&E). The H&E-stained kidney sections were then imaged using the BZ-X710 microscope. Glomeruli (45-75) from five fish/group in three sections per fish, encompassing the vascular pole, were examined for glomerular area and number of nuclei. A minimum of 100 proximal tubule cross-sectional areas were measured for each group.

For TEM observation, zebrafish kidneys (*n*=4 mesonephros/group) were pre-fixed with 2.5% glutaraldehyde in 0.1 M cacodylate buffer (Nacalai Tesque, Kyoto, Japan) at 4°C for 1 h and post-fixed with 1% osmium tetroxide in 0.1 M cacodylate buffer at 4°C for 1 h. The specimens were routinely dehydrated by passing the tissue through a series of solutions with increasing ethanol concentrations and then embedded in Epon 812 epoxy resin (Nisshin EM, Tokyo, Japan). Thick Epon sections were first cut at a thickness of 0.5-1.0 μm using a Reichert Ultracut microtome (Leica, Nussloch, Germany) and routinely stained with Toluidine Blue. The sections were checked and trimmed into glomeruli during the next step. Ultrathin sections (70-80 nm thick) were cut using a diamond knife on the Reichert Ultracut microtome and mounted on copper grids (Veco Grid H100 mesh; Nissin EM, Tokyo, Japan). Ultrathin sections on copper grids were double-stained with uranyl acetate and lead citrate and observed under a transmission electron microscope (JEM 1400 Flash; JEOL, Tokyo, Japan) at an accelerating voltage of 80 kV. For GBM thickness measurement, ten locations were randomly selected for one kidney tissue along the GBM and quantified using ImageJ software (Fiji distribution, version 1.53f51, National Institutes of Health, Bethesda, MD, USA). To assess the thickness of the GBM, ten randomly assigned points in each image from four fish per group were measured, representing the distance between the endothelial cell membrane and the foot process membrane. Foot process width was calculated from a minimum of 20 podocyte foot processes.

### CR

After 20 weeks of overfeeding, adult zMIR/VDBP zebrafish were divided into non-treatment (DN) and CR groups. The DN group was continuously overfed as described previously. The CR group was fed the same amount as the normal feeding group (20 mg/day) for 6 weeks. Body weight, FBG level, kidney tissue weight and GFP leakage were measured at the end of the experiment.

### RNA-seq analysis

Control and DN zebrafish were sacrificed in an ice water bath, and kidney tissues were dissected for subsequent sequencing. RNA isolation, library preparation and sequencing were performed as described previously (Zang et al., 2022a). Briefly, total RNA was extracted and purified from the dissected kidney tissue using TRIzol reagent (Life Technologies) and the RNeasy Mini-prep Kit (QIAGEN). DNase digestion was performed on the column membranes to eliminate DNA contamination. The total RNA purity was determined using an Eppendorf spectrophotometer (BioPhotometer; Eppendorf, Hamburg, Germany). Ribosomal RNA was extracted from the samples using a RiboZero kit (Illumina). The RNA integrity number (RIN) was assessed using an RNA Nano 6000 Assay Kit on a Bioanalyzer 2100 system (Agilent Technologies, CA, USA). Samples with RIN≥8.7 were used for next-generation sequencing library preparation. RNA library construction and Illumina sequencing were conducted by the Novogene Corporation (Beijing, China). In brief, sequencing libraries were generated using the NEBNext Ultra RNA Library Prep Kit for Illumina (New England Biolabs), and index codes were added to attribute sequences to each sample. Clustering of the index-coded samples was performed on a cBot Cluster Generation System using the PE Cluster Kit cBot-HS (Illumina) according to the manufacturer's instructions. After cluster generation, the library preparations were sequenced using the Illumina NovaSeq 6000 platform (Illumina) with a paired-end sequencing length of 150 bp (PE150). Original image data from the Illumina platform were transformed into raw reads by base calling and stored in FASTQ files. Raw reads were filtered to obtain clean reads by removing adapters, polyN sequences and low-quality reads.

### Bioinformatics analysis

Bioinformatics analysis was performed using CLC Genomics Workbench software 20.0.2 (QIAGEN Bioinformatics, Germantown, MD, USA). High-quality sequencing reads were then mapped to the annotated genome of *Danio rerio* to construct GRCz11. The transcript expression values were detected and normalized using the transcripts per million algorithm. The expression data were exported from the CLC Genomics Workbench using Microsoft Excel spreadsheets. According to the Ensembl gene ortholog database (https://www.ensembl.org/biomart/martview), all non-human genes were then converted to human orthologs. The resulting *P*-values were adjusted using the Benjamini and Hochberg approach to control the false discovery rate. DEGs were identified if |fold change|≥1.5 and *P*<0.05. The R package ggplot2 (version 3.5.0; https://ggplot2.tidyverse.org/) and the online biological tool DAVID 6.8 (https://david.ncifcrf.gov/) were used to analyze the molecular and functional characteristics of the DEGs. GO enrichment interactions were further analyzed using ClueGO, a Cytoscape plug-in that displays non-redundant biological terminology for large gene clusters in a functionally grouped network (Bindea et al., 2009). Molecular network and pathway analyses were performed using the IPA software (QIAGEN).

### Comparative transcriptome analysis

The RNA-seq dataset GSE142025 was downloaded from the Gene Expression Omnibus database. After bioinformatics analysis, DEGs for early human DN versus control samples, and advanced human DN versus control samples were extracted using the following conditions: |fold change|>1.5 and *P* <0.05. DEGs from zebrafish DN, early human DN and advanced human DN were used for comparative analysis using the IPA software.

### Metformin treatment

zMIR/VDBP zebrafish were overfed for 8 weeks and proteinuria was measured as described above. The selected DN zebrafish were divided into two groups: (1) DN zebrafish that continued to be overfed for 1 week and (2) DN zebrafish that were administered metformin (Enzo Life Sciences, Farmingdale, NY, USA), as described previously with minor modifications (Zang et al., 2017). In brief, we dissolved metformin in fish water to a final concentration of 50 μM. The metformin solution was freshly prepared and changed daily. FBG levels and proteinuria were measured after 7 days of metformin exposure.

### Western blotting

Kidneys were dissected and quickly homogenized with cold T-PER Tissue Protein Extraction Reagent (Thermo Fisher Scientific) supplemented with Halt Phosphatase Inhibitor Cocktail (Thermo Fisher Scientific). The lysates were centrifuged at 20,000 ***g*** for 15 min at 4°C and supernatants were collected. Protein concentrations were determined using the Pierce 660 nm Protein Assay Kit (Thermo Fisher Scientific). Equal amounts of protein (5 µg/lane) were separated on NuPAGE 4-12% Bis-Tris Gels (Thermo Fisher Scientific) and transferred onto polyvinylidene difluoride (PVDF) membranes (iBlot Transfer Stack; Thermo Fisher Scientific). After blocking for 1 h at room temperature with the PVDF Blocking Reagent for Can Get Signal (Toyobo, Osaka, Japan), the membranes were incubated overnight at 4°C with primary antibodies against Akt (1:2000, #4691, Cell Signaling Technology), pAkt (Ser473) (1:2000, #4060, Cell Signaling Technology) and actin (1:5000, MAB1501, Millipore Bioscience Research Reagents, Bedford, MA, USA) diluted in Can Get Signal 1 (Toyobo). The membranes were then washed and incubated with secondary antibodies – goat anti-rabbit IgG (H&L) (1:10,000, ab6721, Abcam) and goat anti-mouse IgG1 (HRP) (1:10,000, ab97240, Abcam) for 1 h at room temperature. Membranes were probed by ImmunoStar Zeta kit (Fujifilm Wako) and scanned using an Amersham ImageQuant 800 system (Cytiva). The optical densities of immunoreactive bands were analyzed using ImageQuant TL 8.1 software (Cytiva). The protein expression levels of pAkt and Akt were normalized to those of actin.

### Statistical analyses

Results show the mean±standard error of the mean (s.e.m.). All data were analyzed using a two-tailed unpaired *t*-test or one-way ANOVA with the Bonferroni–Dunn multiple comparisons test, depending on the number of comparisons, using GraphPad Prism version 9.3.1 (GraphPad Software, San Diego, CA, USA). Statistical significance was considered as *P*≤0.05.

## Supplementary Material

10.1242/dmm.050438_sup1 Supplementary information
