# Supplementary material for: A zebrafish model of diabetic nephropathy shows hyperglycemia, proteinuria and activation of the PI3K/Akt pathway
Source: Dis Model Mech. 2024 May 29;17(5):dmm050438. doi: 10.1242/dmm.050438 (PMC11152558; doi:10.1242/dmm.050438)
Supplement: Supplementary information [file dmm-17-050438-s1.pdf]

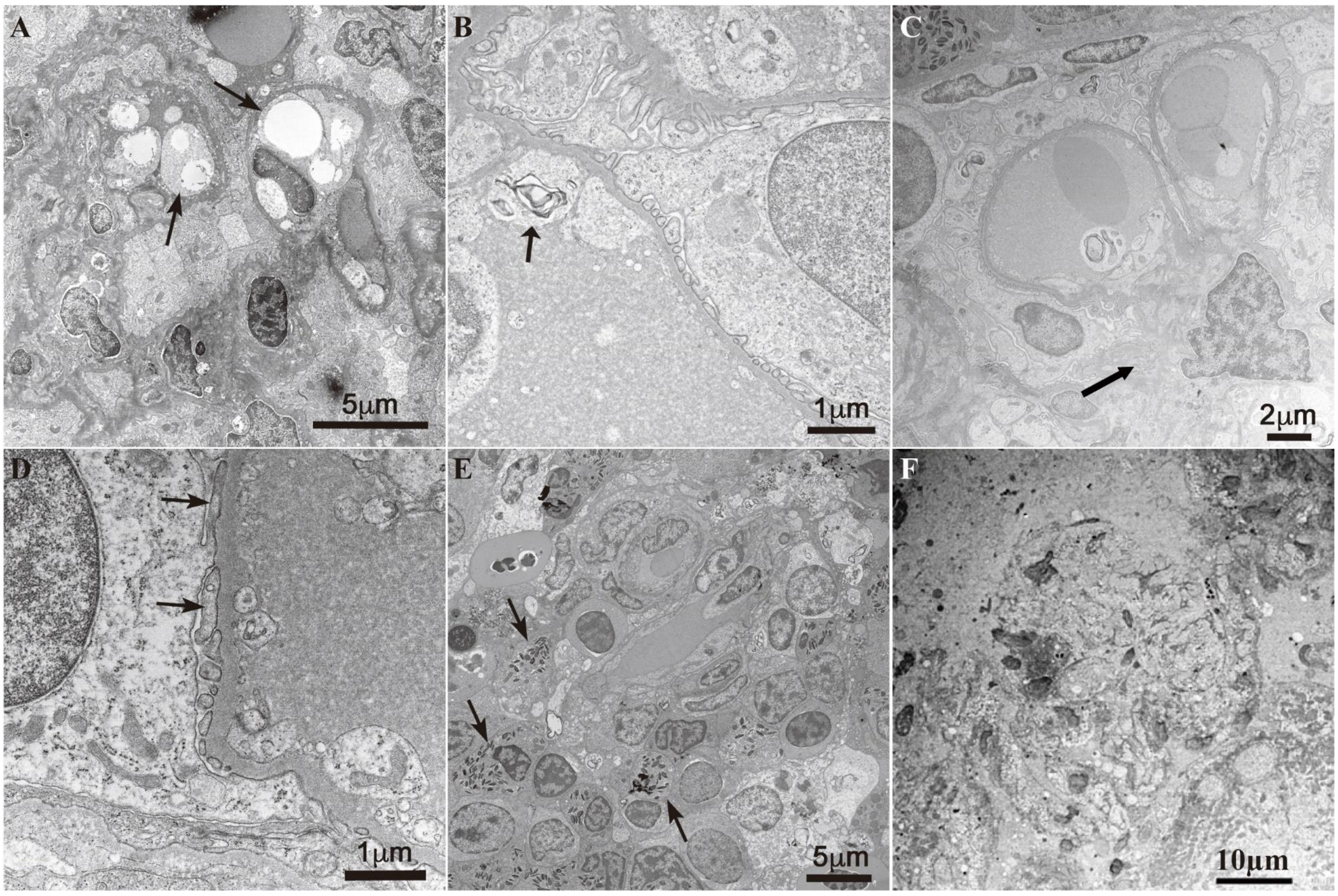

Fig. S1. Overfed zMIR/VDBP zebrafish develop severe renal injury. (A) Endothelial cell sclerosis in glomeruli (black arrow). (B) Mesangial interposition of the glomeruli (black arrows). (C) Mesangial expansion in the glomeruli (black arrow). (D) Podocyte foot process effacement (black arrows). (E) Eosinophilic infiltration (black arrows). (F) Glomerular sclerosis.

Disease Models & Mechanisms • Supplementary information

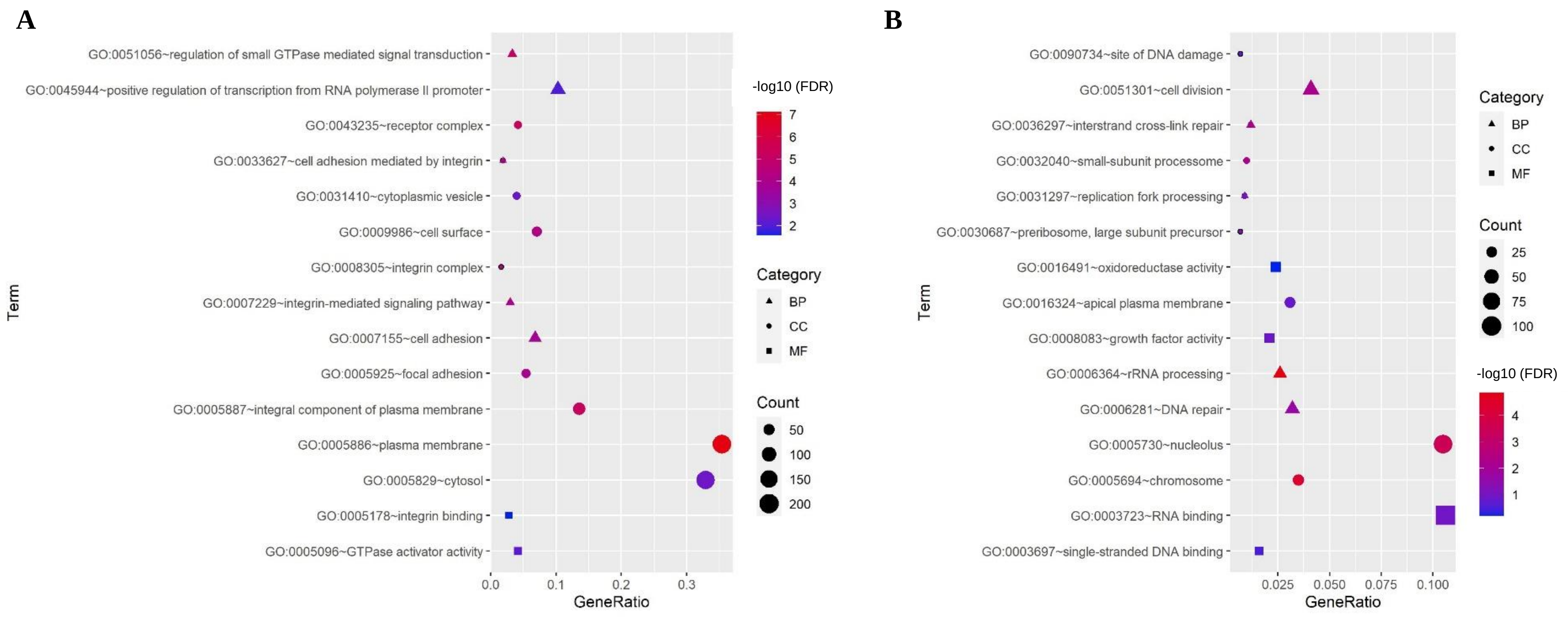

Fig. S2. Gene ontology (GO) enrichment analysis using upregulated and downregulated differentially expressed genes (DEGs) in DN zebrafish. (A) Dot blot showing the top 15 GO-enriched terms using the upregulated DEGs. The y-axis indicates GO terms for biological processes (BP), cellular components (CC), and molecular functions (MF). The X-axis indicates the gene enrichment ratio (gene ratio). The bubble size indicates the number of overlapping genes. The color bar indicates the adjusted FDR value (-log(FDR)); red represents a higher value and blue represents a lower value. (B) Top 15 GO-enriched terms using downregulated DEGs.

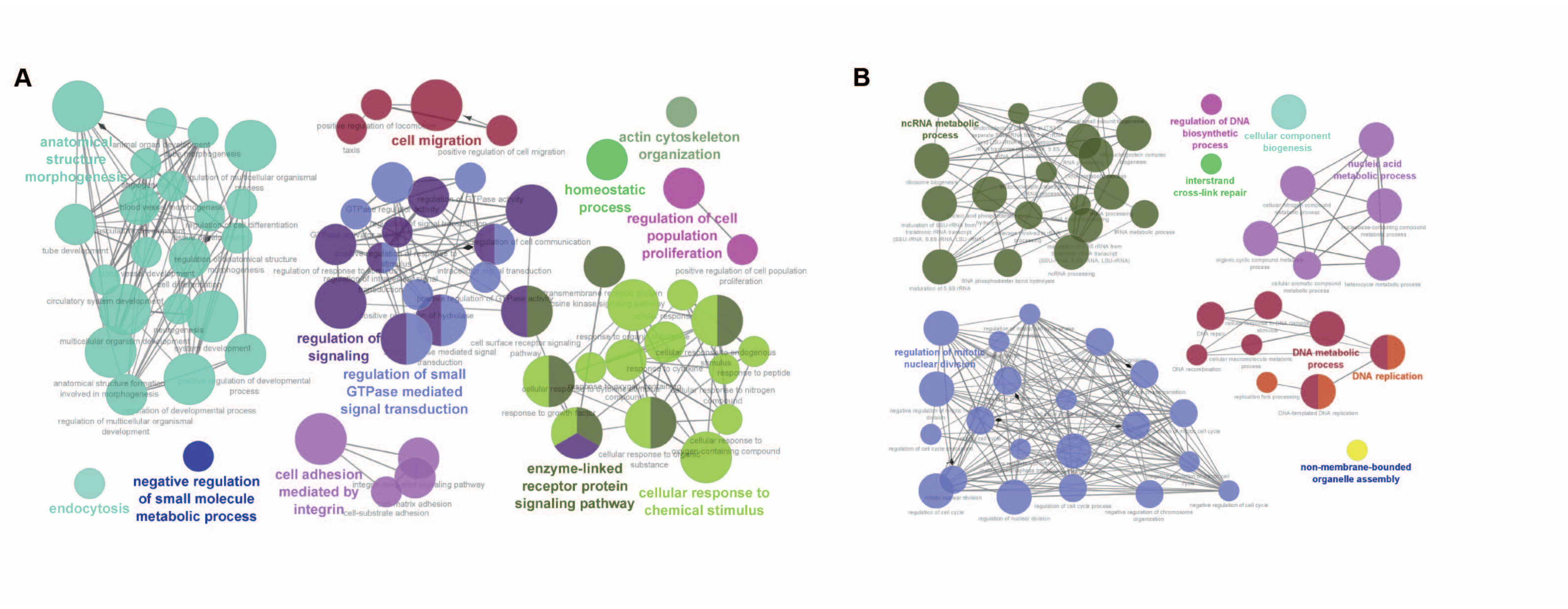

Fig. S3. Functional gene cluster interaction using upregulated and downregulated DEGs in zebrafish with DN. (A) Functional gene cluster interactions of upregulated DEGs in zebrafish with DN. The color and size of the nodes in a cluster correspond to the number of mapped genes for each GO word, and the nodes in the same cluster have the same color. A node label was chosen based on the recurring themes in the processes within the cluster. (B) Functional gene cluster interactions using downregulated DEGs in zebrafish with DN.

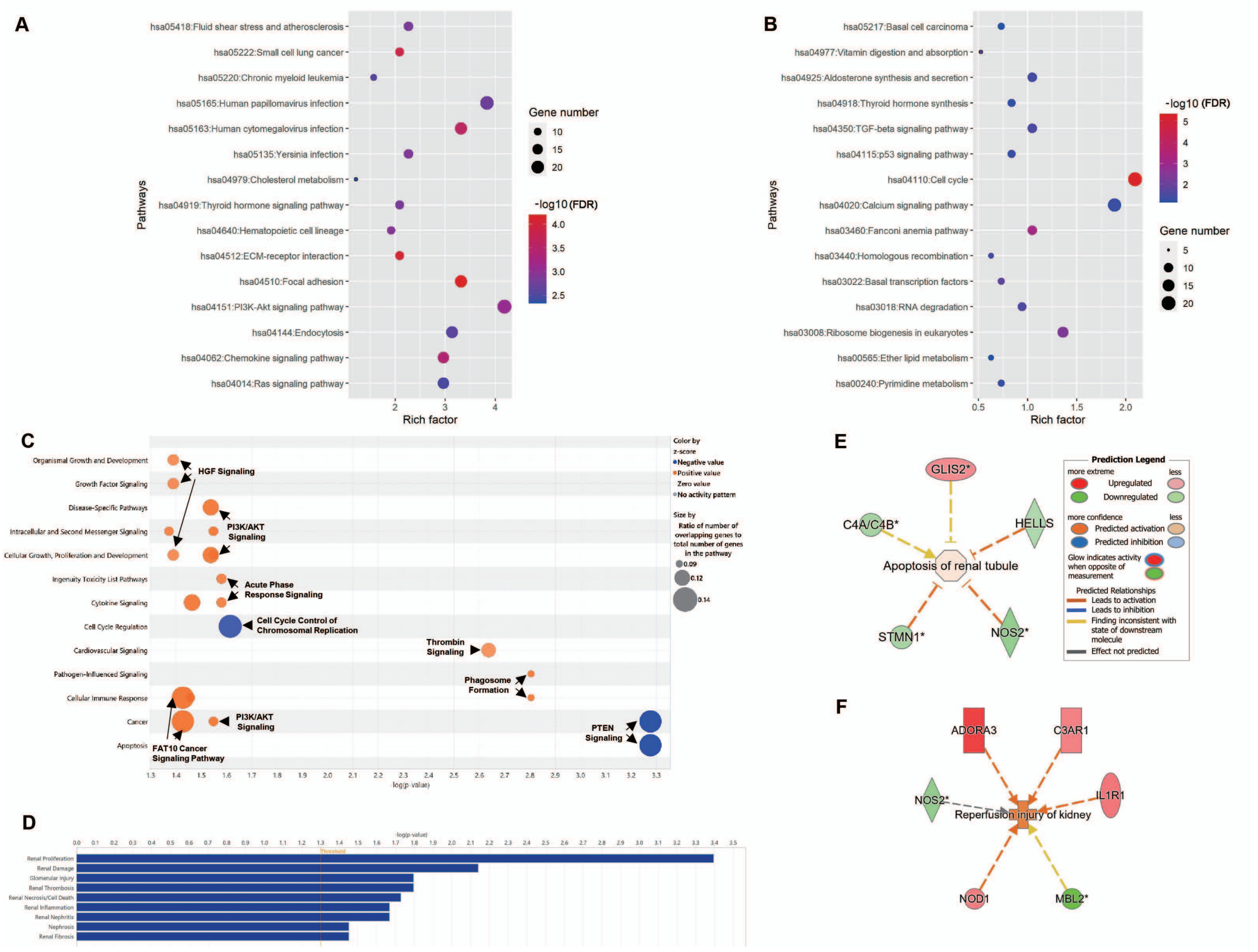

Fig. S4. Bioinformatic analysis using upregulated and downregulated differentially expressed genes (DEGs) in zebrafish with DN. (A) Kyoto Encyclopedia of Genes and Genomes (KEGG) pathway analysis of upregulated DEGs showing the top 15 significantly regulated functional signaling pathways with FDR < 0.05. (B) the top 15 KEGG enrichment pathways among the downregulated DEGs. The Y-axis indicates the KEGG enrichment pathways, and the X-axis indicates the Rich factor, which is the ratio of the DEG number to the total gene number in a certain pathway. The bubble size shows the number of DEGs in the pathway. The color bar indicates the adjusted FDR value (-log(FDR)); red represents a higher value, and blue represents a lower value. (C-F) Ingenuity pathway analysis (IPA) of the DEGs in zebrafish with DN. (C) The bubble chart plots of the regulated canonical pathways in zebrafish with DN were analyzed using all DEGs. The Y-axis on the left indicates the pathway categories, and X-axis shows the negative log of Fisher's Exact Right-tailed p-value (-log(p-value)). The reported pathways were filtered with a z-score > 2 and -log(p-value) > 1.3. The bubble color is determined by their z-score, and the bubble size is related to the ratio of the number of overlapping genes to the total number of genes in the pathway. A negative z-score indicates an inhibited signaling pathway, whereas a positive z-score indicates an activated signaling pathway. (D) Identified "Tox Functions" that are limited to "Nephrotoxicity" analyzed using IPA. The score was cut off by a -log(p-value) > 1.3. (E) Activated "apoptosis of renal tubule" network that belongs to the "Renal Necrosis/Cell Death" category in D. (F) Activated "Reperfusion injury of kidney" network in the "Renal Damage" category in D. Upregulated DEGs are labeled red, and downregulated DEGs are labeled green. An increase in the saturation of color corresponds to an increase in the fold change of the DEGs (for example, a high saturation indicates a large fold change compared to the control group). The lines connecting the genes to the predicted functions represent the relationships, with red representing activation, blue representing inhibition, and gray representing the effect not predicted. For other indicators, please refer to the Prediction Legend.

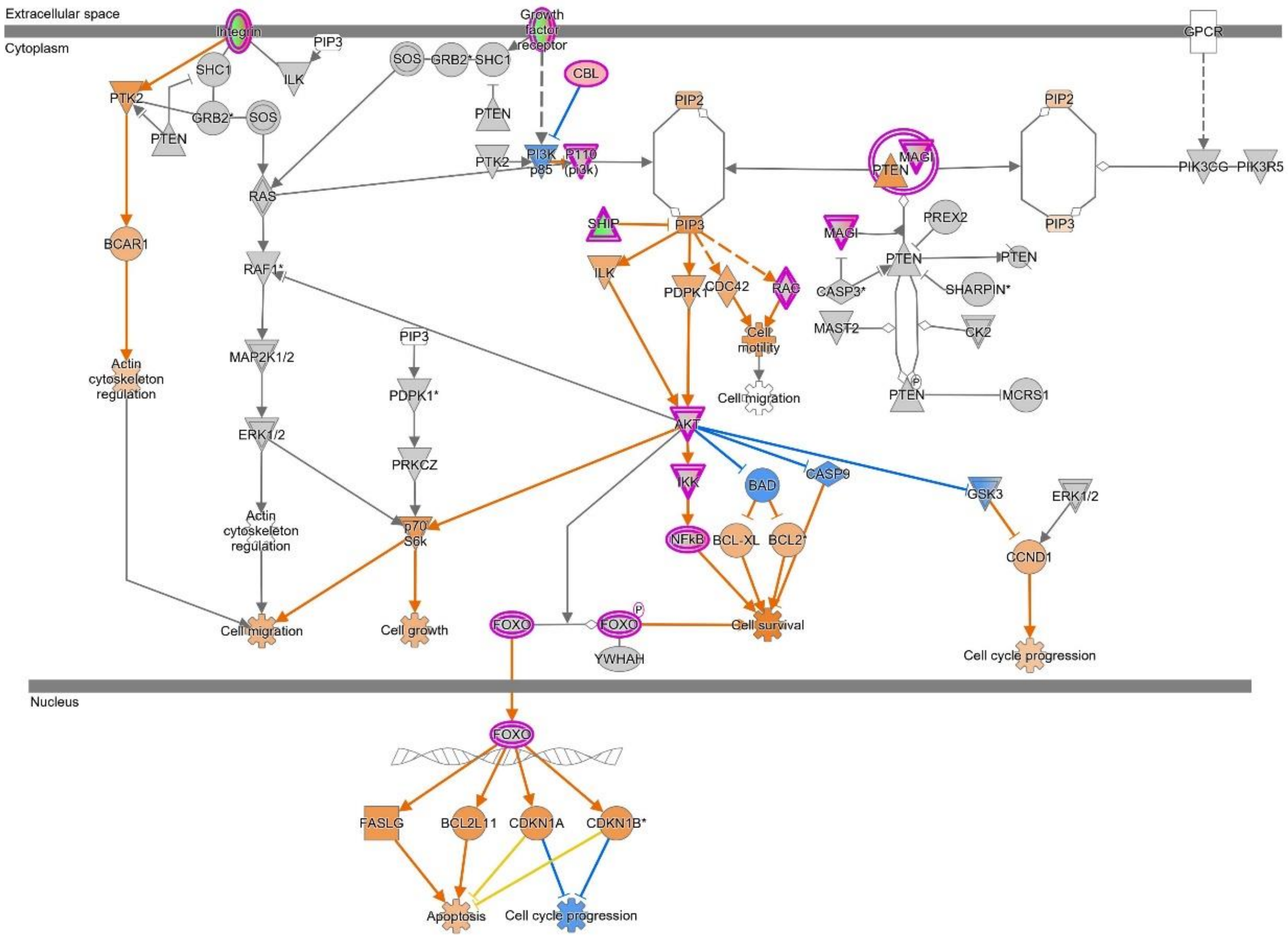

Fig. S5. The inhibited PTEN signaling pathway in DN Zebrafish. Upregulated and downregulated DEGs are shown in red and green, respectively, and the predicted activation or inhibition effects of signaling intermediates and pathways are shown as orange and blue lines, respectively.

Disease Models & Mechanisms • Supplementary information

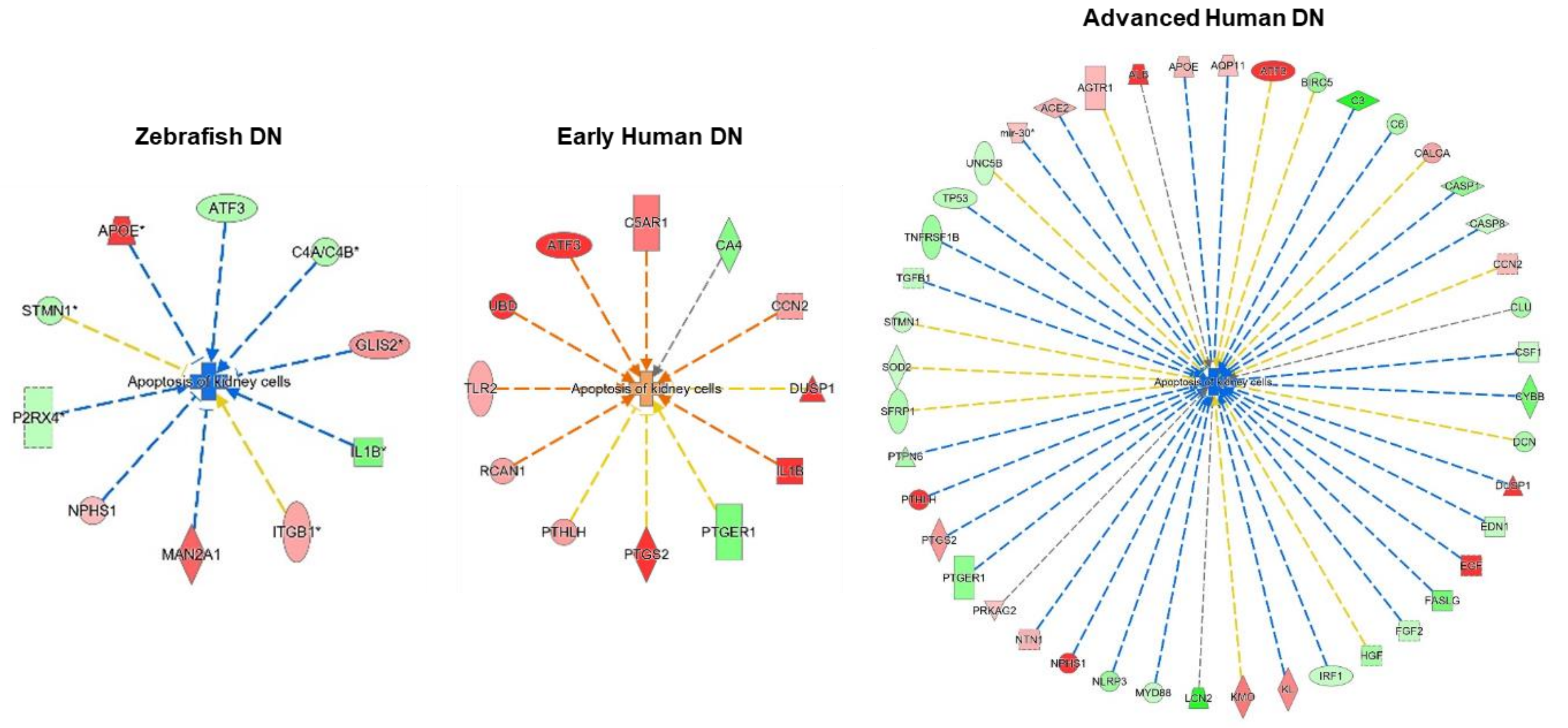

Fig. S6. Comparative transcriptome analysis results between zebrafish DN, early human DN, and advanced human DN. Network details of "Apoptosis of kidney cells" in zebrafish DN, early human DN, and advanced human DN. Upregulated DEGs are labeled red, and downregulated DEGs are labeled green. An increase in the saturation of color corresponds to an increase in the fold change of the DEGs (for example, a high saturation indicates a large fold change compared to the control group). The lines connecting the genes to the predicted functions represent the relationships, with red representing activation and blue representing inhibition.

Disease Models & Mechanisms • Supplementary information

A

Zebrafish DN

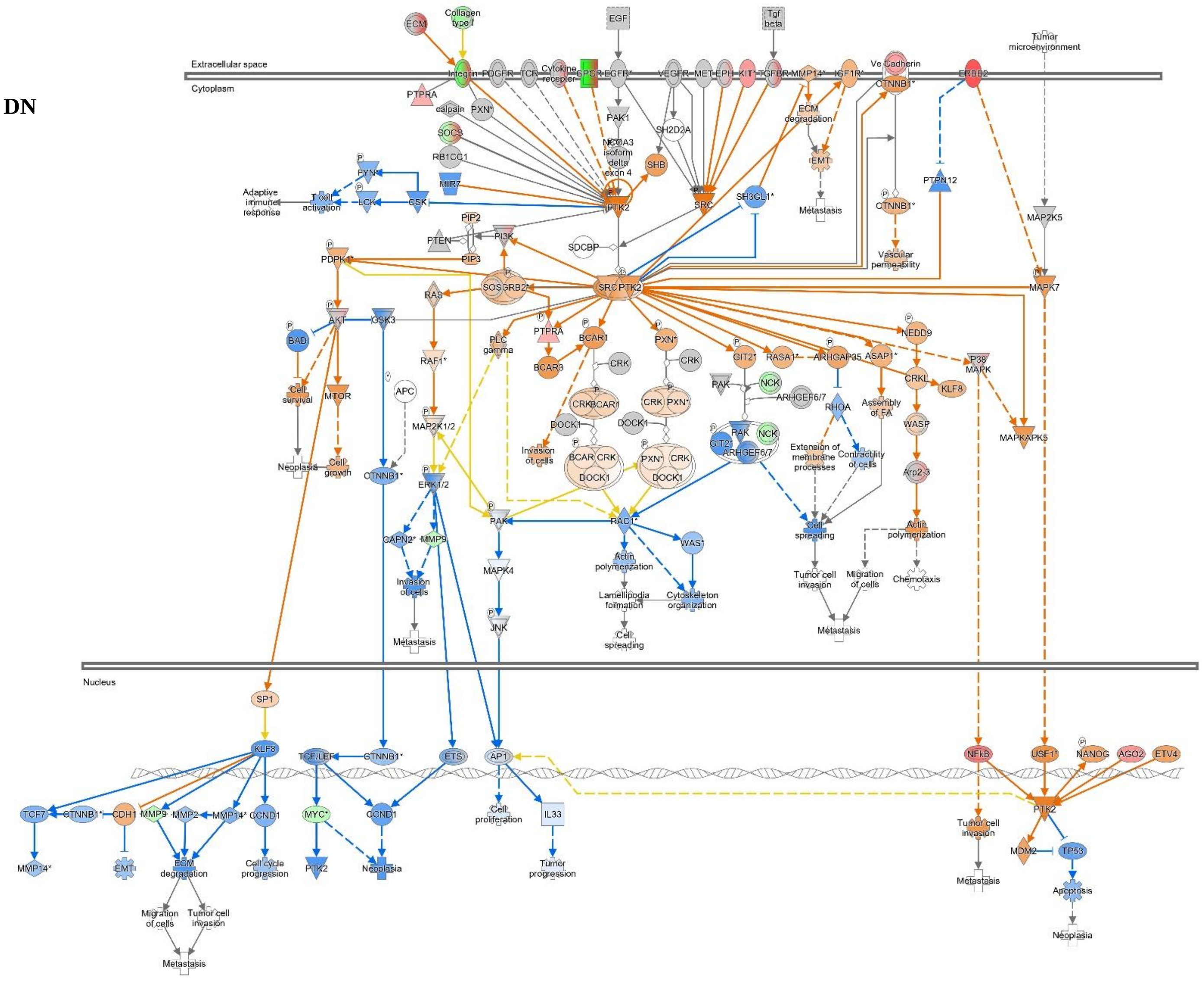

Disease Models & Mechanisms • Supplementary information

B

Early Human DN

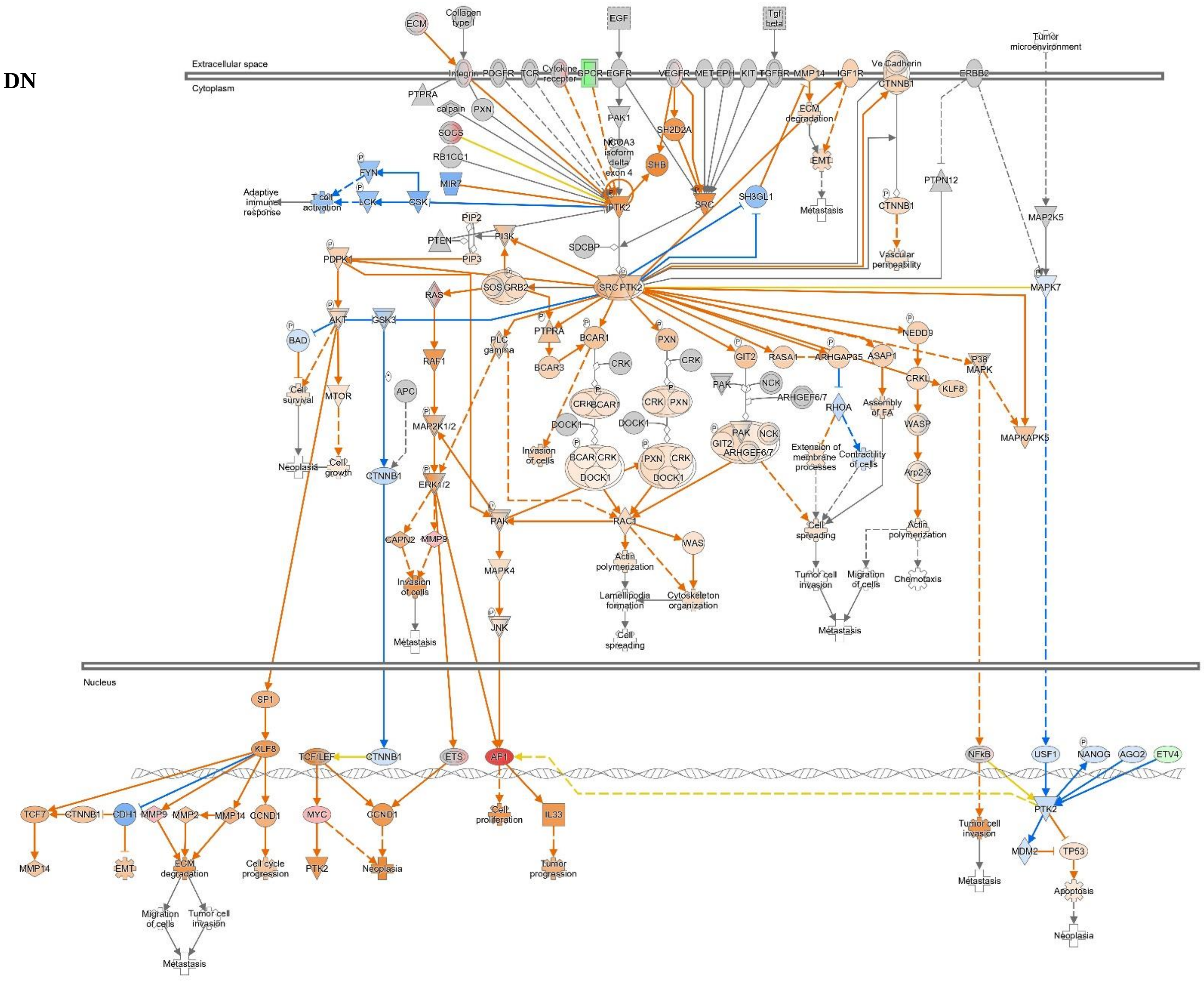

Disease Models & Mechanisms • Supplementary information

C

Advanced Human DN

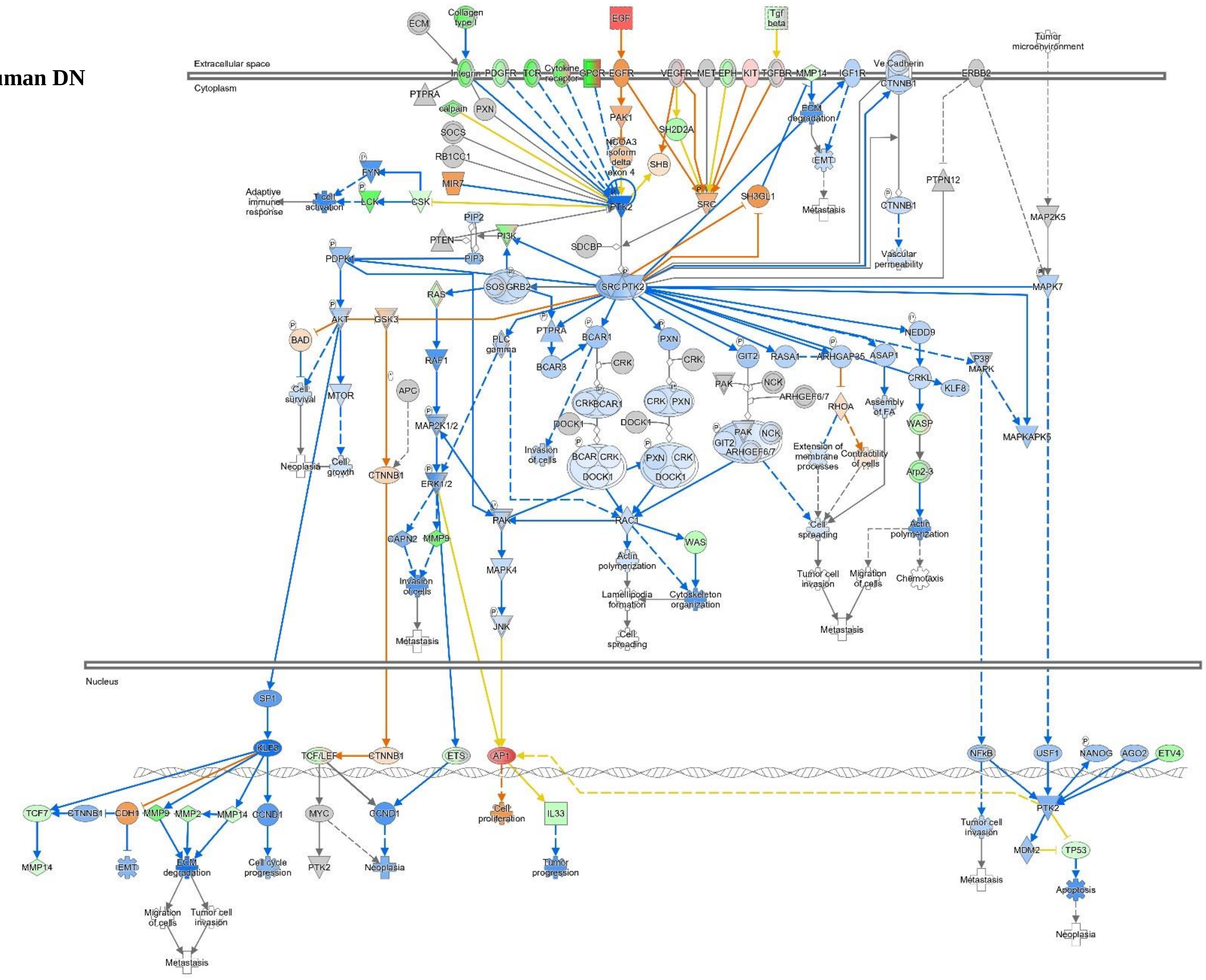

Fig. S7. FAK signaling pathways identified by IPA in the zebrafish DN, early human DN, and advanced human DN. FAK signaling pathway was activated in zebrafish DN (A) and early human DN (B), but inhibited in advanced DN (C). Upregulated and downregulated DEGs are shown in red and green, respectively, and the predicted activation or inhibition effects of signaling intermediates and pathways are shown as orange and blue lines, respectively.

Disease Models & Mechanisms • Supplementary information

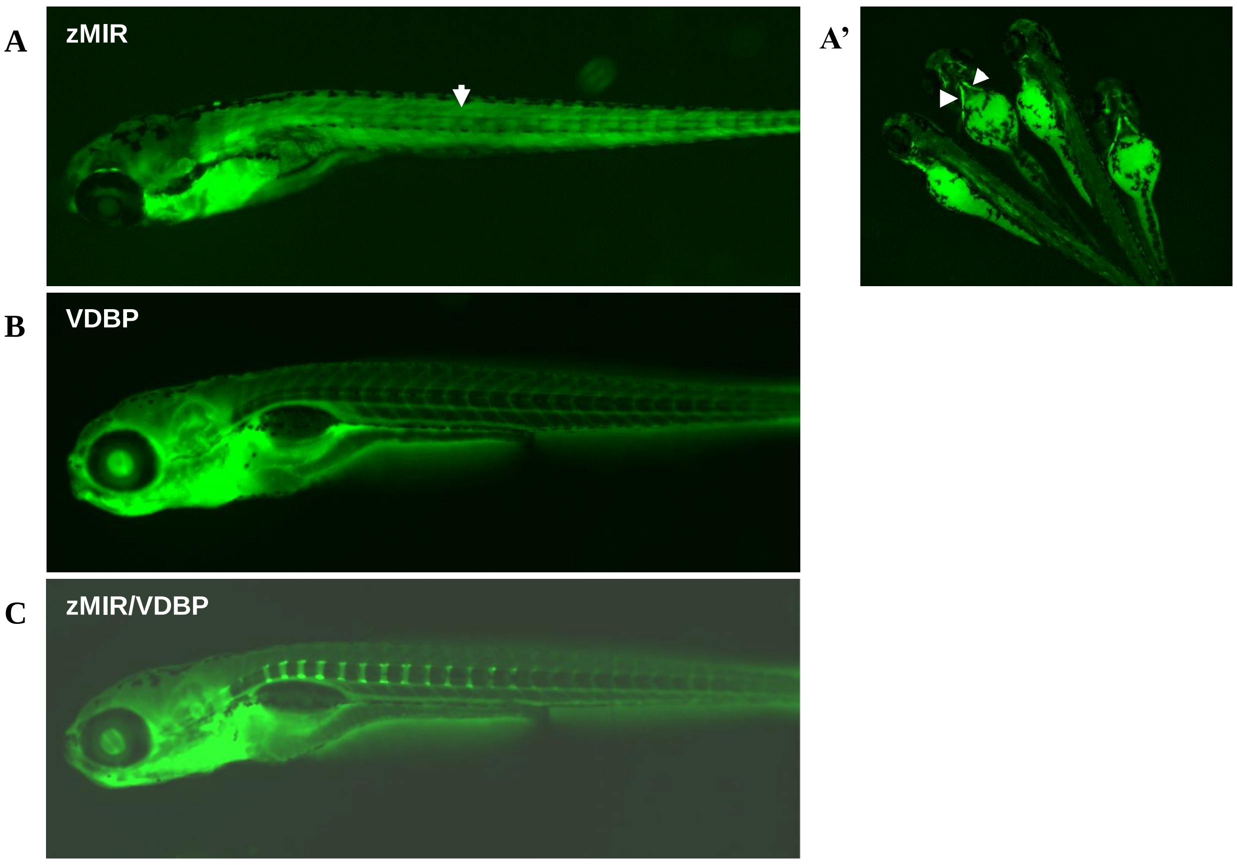

Fig. S8. Different GFP expression patterns in the three transgenic zebrafish strains used in this study. (A) Lateral view of a 5-d-old zMIR zebrafish larva. Specific GFP signals were observed in muscles and jaws (white arrows). (B) Lateral view of 7-d-old VDBP zebrafish larvae. GFP fluorescence is evident in the vasculature. (C) Side view of 7-d-old zMIR/VDBP zebrafish larvae. GFP fluorescence was observed in the jaw, vasculature, and intervertebral discs.

Disease Models & Mechanisms • Supplementary information
